# Characterization of the Biochemical Recurrence Prediction Ability and Progression Correlation of Peroxiredoxins Family in Prostate Cancer Based on Integrating Single‐Cell RNA‐Seq and Bulk RNA‐Seq Cohorts

**DOI:** 10.1002/cam4.70855

**Published:** 2025-04-25

**Authors:** Shan Tang, Jinchuang Li, Weicheng Tian, Yuanfa Feng, Yulin Deng, Zeheng Tan, Zhaodong Han, Huichan He, Yongding Wu, Chuyang Huang, Keping Ning, Feng Liu, Hongwei Luo, Shanghua Cai, Jianheng Ye, Weide Zhong

**Affiliations:** ^1^ Urology Department The Central Hospital of Shaoyang Shaoyang China; ^2^ Department of Urology Guangzhou First People's Hospital, School of Medicine, South China University of Technology Guangzhou China; ^3^ Guangdong Key Laboratory of Clinical Molecular Medicine and Diagnostics Guangzhou First People's Hospital, School of Medicine, South China University of Technology Guangzhou China; ^4^ Guangdong Provincial Key Laboratory of Urology The First Affiliated Hospital of Guangzhou Medical University, Guangzhou Medical University Guangzhou China; ^5^ Guangzhou National Laboratory Guangzhou China; ^6^ Department of Urology The First Dongguan Affiliated Hospital, Guangdong Medical University Dongguan China

**Keywords:** biochemical recurrence, peroxiredoxins, PRDX5, prostate cancer

## Abstract

**Introduction:**

The peroxiredoxins (PRDXs) family plays a crucial role in balancing reactive oxygen species (ROS) levels in tumor cells. However, its potential role in prognosis and therapy response of prostate cancer (PCa) remains unknown.

**Methods:**

In this study, we utilized 2 public single‐cell RNA datasets and 8 bulk‐RNA datasets to investigate the clinical value of six PRDXs family members in PCa. Expression comparison, biochemical recurrence analysis, and therapy response analysis were measured. Pathway enrichments were utilized to predict the potential down‐stream pathway it may involve. In vitro experiments were used to validate the function of PRDX5 in the progression of castration‐resistant prostate cancer (CRPC) cell lines.

**Result:**

Among the PRDXs family, PRDX5 was most related to the advancement of prostate cancer. A nomogram integrating the expression of PRDX5 with clinical features was developed to better predict clinical outcomes in PCa patients compared to 30 published signatures. Immunohistochemistry was used to verify that PRDX5 expression was higher in advanced levels of PCa tissue. Gene Set Enrichment Analysis (GSEA) and pathway predictive analysis revealed that the PRDX5 related genes were mainly relevant to ROS Pathway, Mitochondria‐related functions, cellular respiration, and oxidative phosphorylation. In vitro cell proliferation assays, ROS determination assay, and apoptosis assay together revealed that depletion of PRDX5 induces apoptosis via ROS accumulation in CRPC cells. Moreover, the expression of PRDX5 in CRPC cells also affects the sensitivity to the ARSI therapy.

**Conclusion:**

This study offers new evidence for determining that the expression of PRDX5 is associated with advanced tumor grade, poor prognosis, and suboptimal response to multiple therapies in PCa within the PRDXs family. Last but not least, our study provides new insights into precision medicine in PCa and provides a reference for further research on PRDX5.

## 1 Introduction

Prostate cancer (PCa) is one of the most prevalent cancers in the world, with 299,010 new cases in the United States in 2024 and 1,466,680 new cases globally in 2022 [1, 2]. Most PCa is indolent at the time of diagnosis. Biochemical recurrence (BCR) is a critical stage in the progression of PCa [3]. Approximately 35% of patients experience BCR after receiving radical prostatectomy (RP) or radiation therapy (RT), with elevated prostate‐specific antigen (PSA) levels commonly used as a reference in clinical practice [4]. Once PCa progresses to a more advanced stage, it advances rapidly and significantly increases the mortality rate of patients [5]. Without proper treatment, about 40% of patients will experience prostate cancer‐specific death within 15 years [6]. Drug resistance is a major cause of treatment failure in PCa. Thus, clinicians must assess whether patients require personalized therapy. Exploring a reliable criteria to identify high‐risk patients and the underlying mechanisms of BCR are urgently needed.

Reactive oxygen species (ROS), a wide set of unstable oxygen‐containing molecules, are typical by‐products of cellular metabolism and serve as signaling agents, impacting a range of cellular processes [7]. An alteration in the equilibrium of redox homeostasis, whether due to excessive or inadequate generation of ROS, can have detrimental effects and is associated with several clinical diseases [8, 9]. Elevated ROS levels can inhibit tumor cell growth by inducing oxidative DNA damage, enhancing cell cycle arrest, and promoting apoptosis [10, 11, 12]. The capacity to manage ROS from mitochondrial oxidative metabolism determines the proliferative outcome of cancer cells [13]. In addition, commonly used chemotherapeutic medications exhibit anti‐tumor actions, partially by inducing high levels of ROS [14]. The capacity of cancer cells to adapt to inherent or drug‐induced oxidative stress plays a role in their resistance to chemotherapy and ultimately contributes to the progression of the disease [15, 16].

Peroxiredoxins (PRDXs) are a group of peroxidases that are widely preserved across species and have the capability to remove peroxides, such as hydrogen peroxide (H_2_O_2_), organic hydroperoxides, and peroxynitrite, from the system [17, 18, 19]. The human PRDXs family currently comprises six members which are increasingly recognized as potential therapeutic targets for major diseases, including cancer [20, 21, 22]. The PRDXs family functions as an enzymatic antioxidant defense system within cells, protecting against oxidative and nitrosative damage induced by ROS [23]. Numerous studies have demonstrated that PRDXs family members are altered in various cancer types and are associated with progression and therapy resistance [24, 25, 26, 27]. Nevertheless, the involvement of PRDXs in cancer is intricate. In most solid tumor progression, PRDXs transform their role from oncogenic molecules to cancer protectors by maintaining redox homeostasis within tumor cells [28, 29]. Thus, the role of the PRDX family in prostate cancer needs to be further explored.

In this study, we utilized 2 public single‐cell RNA datasets and 8 bulk‐RNA datasets to investigate the clinical value of six Peroxiredoxin family members in prostate cancer. The study investigated the expression comparison and BCR of the members. After determining PRDX5 as the most significant prognostic factor in the PRDX family, we developed a nomogram integrating PRDX5 expression with clinical features to better predict clinical outcomes in prostate cancer patients than 30 other 30 public gene signatures. PRDX5 was also found to be related to several clinical aspects such as prognosis in various molecular subtypes, responses to chemotherapy, and androgen receptor signaling inhibitor (ARSI) therapy in PCa. Further, immunohistochemistry (IHC) in tissue microarray was used to verify the correlation between PRDX5 and PCa progression. Pathway prediction results indicated that the PRDX5 expression level group was primarily related to Oxidative Phosphorylation, Fatty Acid Metabolism, ROS Pathway, cell cycle, glycolysis, and Peroxisome. In vitro cell proliferation assays, ROS determination assays, and apoptosis assays together revealed that depletion of PRDX5 induces apoptosis via ROS accumulation in CRPC cells. Moreover, the expression of PRDX5 in CRPC cells also affects the sensitivity to ARSI therapy. In conclusion, the objective of our study is to determine the value of PRDX5 as a biomarker influencing prognosis and response to treatment for prostate cancer.

## 2 Materials and Methods

### 2.1 Data Source and Procession

RNA‐sequencing data from 100 normal prostate tissue samples were retrieved from the Genotype‐Tissue Expression (GTEx, https://gtexportal.org/). Seven independent public datasets (including TCGA, CancerMap (GSE94767), DKFZ, GSE54460, GSE70769, Cambridge (GSE70768) and GSE116918) were accessed from The Cancer Genome Atlas (TCGA, http://portal.gdc.cancer.gov/) and Gene Expression Omnibus (GEO, https://www.ncbi.nlm.nih.gov/geo/). Single‐cell sequencing analysis data were obtained from the sequencing results of GSE157703 and GSE141445 in GEO. Information on 545 drugs from the Cancer Therapeutics Response Portal (CTRP) database and 198 drugs from the Genomics of Drug Sensitivity in Cancer (GDSC) database was retrieved. Furthermore, data pertaining to ARSI therapy cohorts were obtained from Github (https://github.com/cBioPortal/datahub/tree/master/public/prad_su2c_2019).

To further validate the predictive performance of the PRDX5‐Nomo signature, the C‐indexes and hazard ratios (HR) of 30 gene expression prognostic signatures trained with the robust Cox‐Ridge algorithm were downloaded from the PCaDB database (http://bioinfo.jialab‐ucr.org/PCaDB/) [30]. We then computed the C‐index and HR for each prognostic signature in these cohorts.

### 2.2 Gene Alteration Analysis

In order to look for somatic mutations linked to PRDXs, we used the cBioPortal website for our investigation (https://www.cbioportal.org), generating waterfall plots and bar charts. Additionally, we performed a copy number variation (CNV) analysis of PRDXs using the R package “Maftools,” which produced lollipop plots and Circos plots. These visualizations illustrate the mutation distribution of PRDXs in TCGA prostate cancer patients.

### 2.3 Single‐Cell RNA‐Seq Data Analysis

We analyzed the single‐cell sequencing data using the “Seurat” package. Firstly, in the GSE157703 dataset, data quality control (QC) was conducted by retaining cells with less than 40% mitochondrial gene content and genes expressed in at least three cells within a range of expression greater than 200. In the GSE141445 dataset, QC was performed by retaining cells with less than 20% mitochondrial gene content and genes expressed in at least three cells within a range of expression between 200 and 5218. Subsequently, highly variable genes were identified for further analysis, with the number of highly variable genes set to 2000. Batch effects within individual sample data were corrected using the “Harmony” package. Cell clusters were constructed using the “FindClusters” and “FindNeighbors” functions, and visualized using the “Umap” method. Finally, cell annotation based on marker genes for different cell types was performed.

### 2.4 Survival Analysis and Construction of a Predictive Nomogram

Patients from datasets were categorized into high‐expression and low‐expression groups based on the optimal cutoff value of PRDXs gene expression levels. Subsequently, we conducted KM curve analysis using the “survminer” package in R to investigate whether there were significant differences in BCR between the high‐expression and low‐expression groups. Furthermore, the “timeROC” program was used to conduct ROC curve analysis to assess the sensitivity and specificity of PRDXs in predicting BCR in PCa patients. We also compared the area under the curve (AUC) of PRDXs with other clinical features. Furthermore, we explored the correlation between PRDX5 and several clinical features, including Gleason score and Pathological T stages. Univariate and multivariate Cox regression analyses were conducted on the TCGA‐PRAD dataset to determine if PRDX5 is an independent prognostic factor for predicting survival in PRAD patients. In order to enhance the prognostic precision and prediction capacity of our model, we created a nomogram that measures the predicted survival of PRAD patients by combining PRDX5, Gleason score, and Pathological T stages [31]. Finally, we evaluated the accuracy and precision of the nomogram using ROC curves, the C‐index, and calibration curves, as well as assessing its net clinical benefit using decision curve analysis.

### 2.5 PAM50 Clustering

PAM50 clustering was performed based on the original algorithm by Parker et al. [32]. The source code was downloaded from the University of North Carolina Microarray Database.

### 2.6 Drug Sensitivity Analysis

To achieve personalized therapy, R package “oncoPredict” was utilized to predict chemotherapy sensitivity in prostate cancer patients from TCGA. To attempt to determine the half‐maximal inhibitory concentration (IC50), “oncoPredict” matches the tissue gene expression patterns of patients to the expression profiles of cancer cell lines. The relationship between each patient's level of PRDX5 expression and each medication was assessed using Spearman correlation, with *p* < 0.05 being regarded as statistically significant.

### 2.7 Immunohistochemistry Staining Analysis

We accessed IHC staining images of PRDX5 in normal prostate tissue, low‐grade, and high‐grade prostate cancer tissue from the Human Protein Atlas database to preliminarily identify the expression pattern of PRDX5.

Subsequently, we employed IHC to detect the expression of PRDX5 (67599‐1‐Ig, Proteintech) in Chinese tissue microarrays (HProA150CS01, OUTDO BIOTECH), employing adjacent non‐tumor prostate tissue and tumor tissues of different pathological T‐stages of prostate cancer. Following staining, we quantified staining intensity scores and generated box plots and violin plots for analysis. The criteria used to score the staining of the tissue samples were based on the percentage of cells stained: 0% stained cells, score 0; 1%–10% stained cells, score 1; 11%–50% stained cells, score 2; 51%–80% stained cells, score 3; and 81%–100% stained cells, score 4. The criteria used to score the tissue sample staining based on intensity were as follows: no staining, score 0; weak staining intensity, score 1; moderate staining intensity, score 2; high staining intensity, score 3; and strong staining intensity, score 4.

### 2.8 Functional Enrichment Analysis

We performed a differentially expressed gene (DEGs) analysis comparing the high and low PRDX5 expression groups using the “DESeq2” software and conducted Gene Set Enrichment Analysis (GSEA) based on the method described in past research [33]. This analysis adhered to stringent criteria, with qvalue < 0.05 and |NES| > 1, employing the R package “clusterProfiler” for pathway annotation and enrichment. Then, we conducted Pearson correlation analysis to identify the genes most closely related with PRDX5 expression in the TCGA_PRAD dataset with a filtering criterion of |cor| > 0.6. Enrichment analyses were performed to elucidate the biological significance of PRDX5 using these genes.

### 2.9 Cell Lines

Human PCa cell lines 22Rv1 and C4‐2B were obtained from the American Type Culture Collection (ATCC HTB‐81 and ATCCCRL1435, Manassas, VA, USA). All cell lines were grown in Roswell Park Memorial Institute 1640 medium (MA0215, Meilunbio, Dalian, China) containing 10% fetal bovine serum (HF1032‐05, Holocene, Guangzhou, China) and 1% penicillin–streptomycin solution (15140‐122, Gibco, Grand Island, NY, USA). Cells were maintained at 37°C with 5% CO_2_.

### 2.10 Cell Proliferation Assay

Cell proliferation was measured by CCK‐8 (Cell Counting Kit‐8, MA0218, Meilunbio) assay and colony formation assay. 5000 cells per well of 22Rv1 and 3000 cells per well of C4‐2B were seeded in 96‐well plates for CCK‐8 assay. 800 cells per well of all cell lines were seeded in 12‐well plates for colony formation assay. Abiraterone (S2246, Selleck) and Enzalutamide (S1250, Selleck) were used in the drug sensitivity assay.

### 2.11 RNAi Transfection of 22Rv1 and C4‐2B Cell Lines

Human PRDX5‐specific siRNA and negative control siRNA were purchased from Genepharma Co. Ltd. (Shanghai, China). The siRNA sequences are shown as follows.

**Table:** 

siRNA		
PRDX5		5′–3′
si‐1	Sense	GAGUGUUAAUGAUGCCUUU(dT)(dT)
	Antisense	AAAGGCAUCAUUAACACUC(dT)(dT)
si‐2	Sense	CCCUGGAUGUUCCAAGACA(dT)(dT)
	Antisense	UGUCUUGGAACAUCCAGGG(dT)(dT)
si‐3	Sense	CAGAGCUGUUCAAGGGCAA(dT)(dT)
	Antisense	UUGCCCUUGAACAGCUCUG(dT)(dT)
si‐NC	Sense	UUCUCCGAACGUGUCACGUTT
	Antisense	ACGUGACACGUUCGGAGAATT

### 2.12 Western Blot Assay

The lysis products were quantified using the BCA Protein Assay Kit (#23227, Thermo Scientific). The protein expression level of the target gene was measured and quantified by western blot analysis according to the protocol of our previous studies [34]. Antibodies applied in the assay are in the dilution as follows: β‐Actin (1:5000; ab8227, Abcam, Cambridge, UK), PRDX5 (1:1000; 17724‐1‐AP, Proteintech).

### 2.13 Determination of Reactive Oxygen Species (ROS) Levels

The levels of intracellular ROS were monitored by using a ROS assay kit (Beyotime, China). Briefly, cells were collected and stained with 10 μM DCFH‐DA at 37°C for 30 min. ROS levels were measured by flow cytometry (BD FACSVerse, BD Biosciences, Franklin Lakes, NJ, USA) and a fluorescent microscope.

### 2.14 Apoptosis Assay

Cells were harvested after different treatments. After that, cells were stained with Annexin V‐APC and 7‐AAD (AP105, MultiSciences, Hangzhou, China). Finally, cells were subjected to a flow cytometer (BD FACSVerse, BD Biosciences, Franklin Lakes, NJ, USA). Analysis was conducted with FlowJo software. The ROS scavenger N‐acetylcysteine (NAC) was bought from MedChemExpress (HY134495). The cells in the NAC group were pretreated with 3 mM NAC for 1 h.

### 2.15 Statistical Analysis

All statistical analyses were performed using R software (version: R 4.3.2). The Wilcoxon test was utilized as a non‐parametric method to estimate differences between two non‐normally distributed variables.

## 3 Results

### 3.1 Landscape of the Genome Alteration Features of the Peroxiredoxin Family in PCa

**FIGURE 1:**
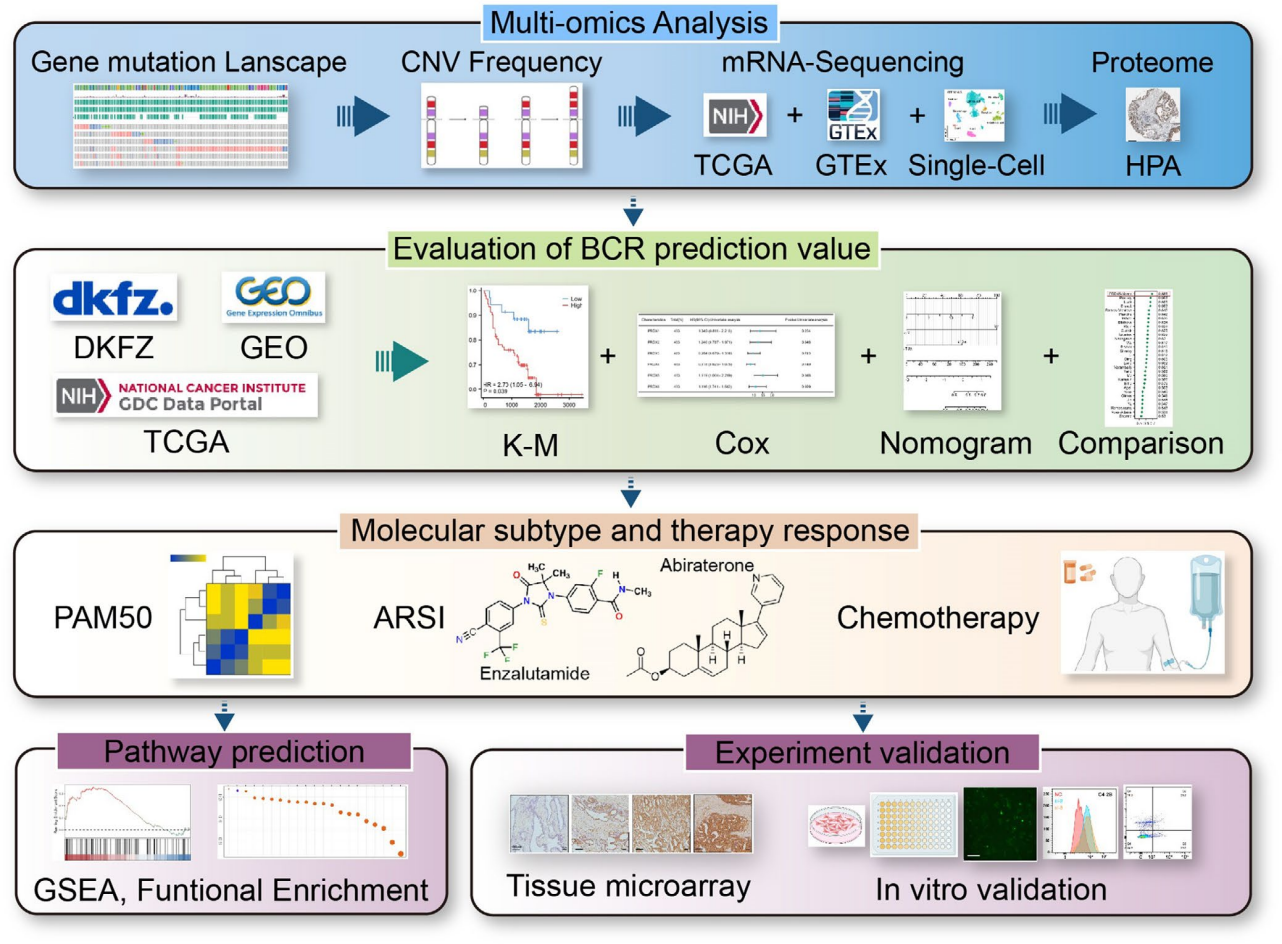
The overview flowchart of our study.

**FIGURE 2:**
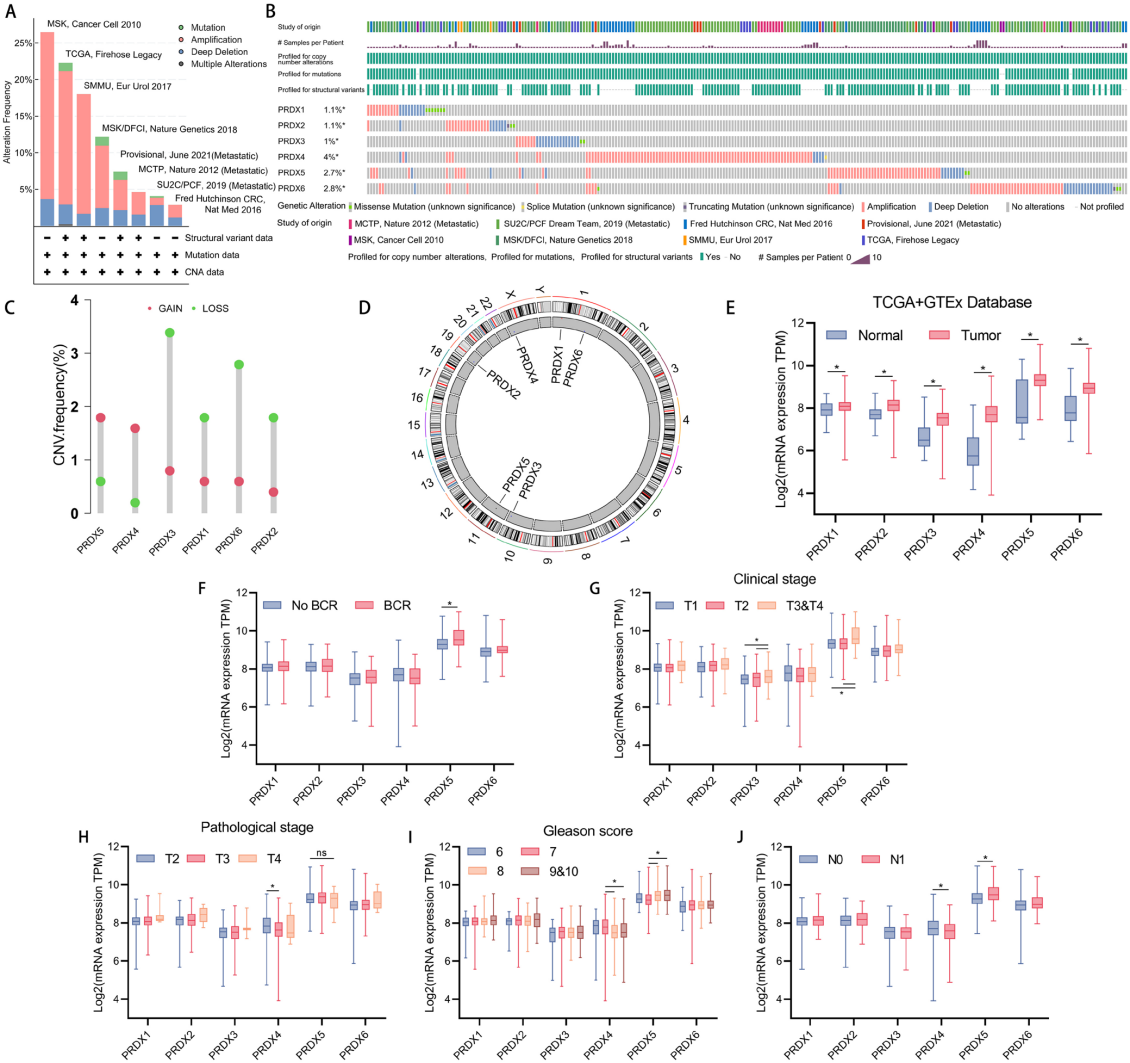
Landscape of the genome and transcriptome alteration features of the peroxiredoxin family in PCa. (A) The bar chart illustrates the alteration proportion around different public databases. (B) The oncoprint plot depicts the genome alteration frequency of each peroxiredoxin across multiple databases. (C) The CNV mutation frequency of 6 peroxiredoxins in the TCGA database. Green and red are for deletion and amplification, respectively. (D) Circus plot of the human genome shows CNV site of PRDXs in PCa. (E) The box plot displays the gene expression levels of PRDXs between tumor tissue samples and normal tissue samples from the TCGA and GTEx databases. (F–J) The box plot displays the gene expression levels of PRDXs in the TCGA database across different BCR statuses (F), Clinical stages (G), Pathological T stages (H), Gleason scores (I), and Clinical N stages (J). (*p < 0.05, **p < 0.01).

A total of 6 main types of peroxiredoxin from published papers were included in our study. Figure 1 depicts the workflow of the research. Copy number alterations (CNAs) are a prominent kind of genetic modification in human cancer and play a crucial role in the advancement of cancer, particularly in prostate cancer [35]. Germline genetic testing and tumor‐directed somatic sequencing hold significant potential for treatment and assessing familial risk, which can aid in guiding treatment decisions [36]. We found that amplifications in PRDXs occupied a larger change frequency across multiple databases (MSK, TCGA, SMMU, MSK/DFCI, Provisional, MCTP, SU2C/PCF, and Fred Hutchinson CRC) using cbioportal (Figure 2A). After integrating data from 8 databases, PRDX4 was found to have the highest alteration frequency (4%), followed by PRDX6 (2.8%) and PRDX5 (2.7%), while PRDX1 (1.1%) and PRDX2 (1.1%) had the lowest frequency (Figure 2B). Additionally, in TCGA, PRDXs exhibited distinct CNV frequencies, with PRDX5 showing the highest CNV frequency amplifications (Figure 2C,D).

### 3.2 Landscape of the Bulk mRNA Expression Features of the Peroxiredoxin Family in PCa

Following that, the mRNA expression of PRDXs was examined by combining data from the TCGA database (483 prostate cancer tissues and 51 adjacent non‐cancerous tissues) and the GTEx database (100 benign prostate tissues). Compared to benign prostate tissues, prostate cancer tissues demonstrated significantly elevated expression levels of PRDXs (*p* < 0.05) (Figure 2E). The expression level of PRDX5 were higher in patients with BCR compared to those who did not (Figure 2F, *p* < 0.05). Furthermore, in patients clinically staged as T3&T4 compared to T1, and T3&T4 compared to T2, PRDX3 and PRDX5 exhibited higher expression levels (Figure 2G, T3&T4 vs. T1, *p* < 0.05; T3&T4 vs. T2, p < 0.05). Patients of pathological stage T3 and T4 exhibited higher PRDX5 levels compared to patients staged as T2, although the difference was not statistically significant. The expression of PRDX4 in patients with pathological stage T2 was significantly higher than in T3 (Figure 2H, *p* < 0.05). Interestingly, compared to patients with Gleason scores of 8 and 9&10, patients with Gleason score of 7 exhibited higher expression levels of PRDX4, while PRDX5 showed the opposite trend, with higher expression levels in patients with higher Gleason scores (Figure 2I, Gleason score = 8 vs. 7, *p* < 0.05; Gleason score = 9&10 vs. 7, p < 0.05). Regarding lymph node staging, PRDX4 showed higher expression levels in patients staged as N0 compared to N1, while PRDX5 exhibited higher expression levels in patients with lymph node metastasis (Figure 2J, *p* < 0.05).

### 3.3 Tissue Sublocalization Expression of Peroxiredoxin Family in PCa

**FIGURE 3:**
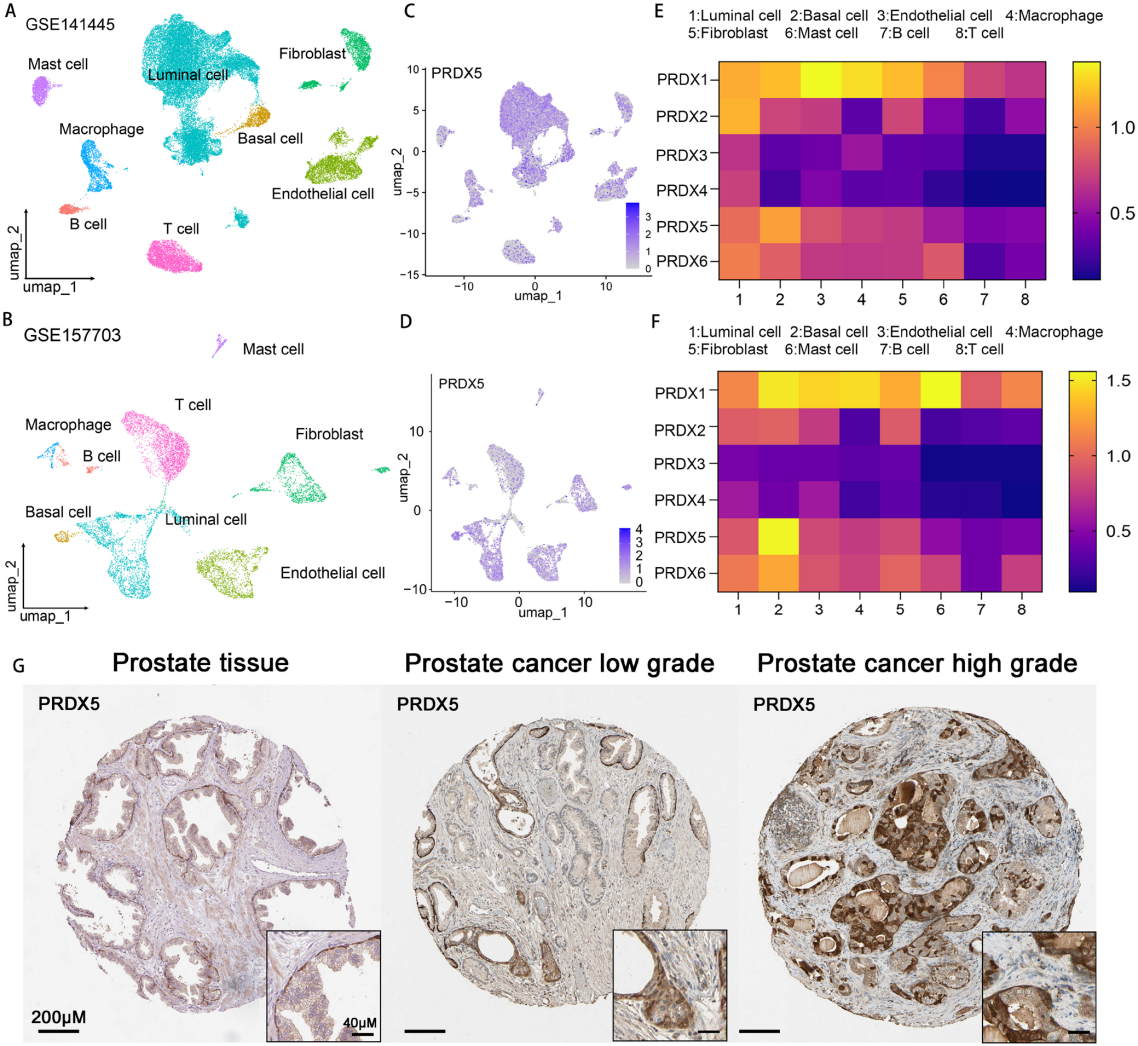
Tissue sublocalization expression of peroxiredoxin family in PCa. (A, B) UMAP of GSE141445 and GSE157703 demonstrates the distribution patterns of subgroups in each dataset annotated by cell type annotation and colored accordingly. (C, D) UMAP plot shows the distribution patterns and the relative expression level of PRDX5 in each dataset. (E, F) The heatmap illustrates the expression levels of PRDXs across different cell types in the GSE141445 and GSE157703 datasets. (G) Immunohistochemistry staining of PRDX5 in Human protein atlas database.

To further elucidate the role of PRDXs in prostate cancer, we performed studies utilizing two single‐cell datasets collected from the GEO database (30,000 cells from GSE141445 and 7000 cells from GSE157703), comprising a total of 15 single‐cell sequencing data samples of prostate cancer tissues. Uniform Manifold Approximation and Projection (UMAP) plots demonstrated effective separation of prostate cancer tissue samples into 8 distinct cell clusters, including luminal cells, basal cells, endothelial cells, macrophages, fibroblasts, mast cells, B cells, and T cells (Figure 3A,B). The markers identified by the differences in the main cell types were visualized as a bubble plot (Figure S1). Notably, all members of the PRDX family exhibited significantly higher expression levels in luminal cells and basal cells in accordance with the UMAP and heatmap (Figure 3C, Figure S2). Regarding the proteomics, IHC staining analysis of prostate tissue or PCa tissue from the Human Protein Atlas database showed that higher staining of PRDX3 and PRDX5 was found in higher grade of prostate cancer (Figure 3G, Figure S3).

### 3.4 Relationship Between mRNA Levels of Peroxiredoxin Family and Prognosis in Prostate Cancer

**FIGURE 4:**
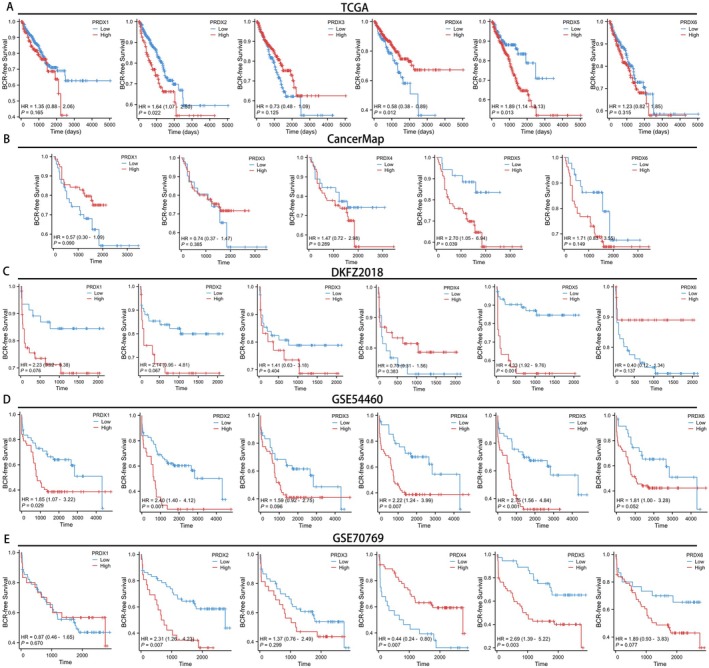
The BCR‐free survival curves in 5 public databases. (A, B) The Kaplan–Meier curves illustrate the biochemical recurrence status of prostate cancer patients across TCGA (A), CancerMap (B), DKFZ2018 (C), GSE54460 (D), GSE70769 (E), stratified by best cutoff grouping based on PRDXs expression levels.

To comprehensively evaluate the prognostic significance of PRDXs in prostate cancer, we generated BCR‐free survival curves for PRDXs across 7 databases (TCGA, CancerMap, DKFZ2018, GSE54460, GSE70769, Cambridge and GSE116918). In the TCGA database, patients with high expression levels of PRDX2 and PRDX5 increased a tendency towards BCR, with respective *p*‐values of 0.022 and 0.013. On the contrary, higher expression of PRDX4 tends to decrease the likelihood of BCR (Figure 4A, *p* = 0.012). In the CancerMap and DKFZ2018 databases, only patients with high expression levels of PRDX5 were more prone to BCR, with respective p‐values of 0.039 and < 0.001 (Figure 4B,C). In the GSE54460 database, patients with high expression levels of PRDX1, PRDX2, PRDX4, and PRDX5 showed a tendency towards increased likelihood of BCR, with respective p‐values of 0.029, 0.001, 0.007, and < 0.001 (Figure 4D). In the GSE70769 database, patients with high expression levels of PRDX2 and PRDX5 exhibited a tendency towards increased probability of BCR, with respective p‐values of 0.007 and 0.003. Consistent with the result from TCGA, higher expression of PRDX4 tends to decrease the probability of BCR (Figure 4E, *p* = 0.007). In the Cambridge dataset, patients with high expression levels of PRDX2 exhibited worse prognosis (Figure S4A, *p* = 0.008). In the GSE116918 dataset, patients with low expression levels of PRDX6 exhibited worse prognosis (Figure S4B, *p* = 0.015). Notably, across all 7 databases, patients in 5 datasets with high expression of PRDX5 were more likely to reach BCR, indicating that PRDX5 is the most promising biomarker within the PRDX family associated with BCR in prostate cancer.

### 3.5 Identification of PRDX5 as the Reliable Prognostic Biomarker for Prostate Cancer Within PRDX Family

**FIGURE 5:**
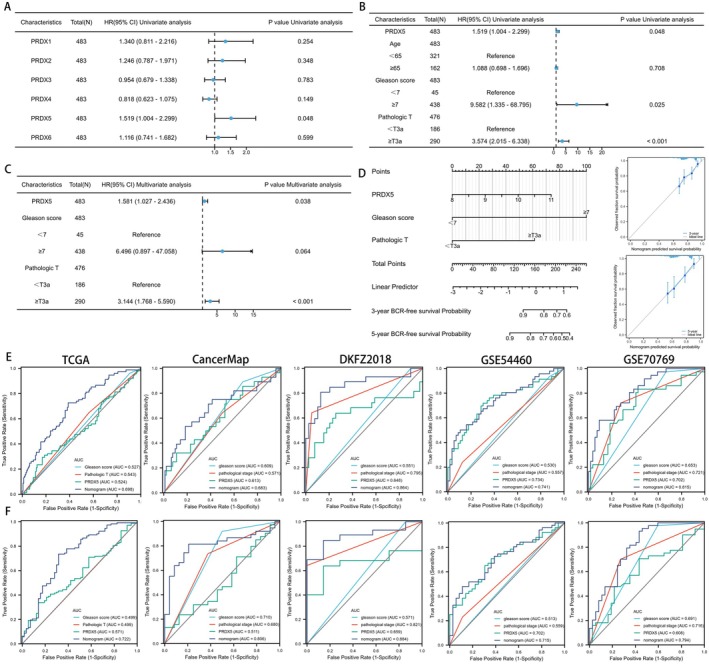
Assessing the predictive significance of PRDXs in conjunction with clinical characteristics in PCa. (A, B) Univariate analyses of PRDXs (A) and clinical characteristics (B) in prostate cancer patients from TCGA. (C) Multivariate analyses of PRDX5 and clinical characteristics for BCR in prostate cancer patients from TCGA. (D) The nomogram and calibrated curve based on PRDX5, Gleason score, and Pathological T stages, along with calibration curves for 3‐year and 5‐year BCR. (E) ROC curves illustrate the predictive performance of Gleason score, Pathological T stages, PRDX5, and the nomogram for 3‐year BCR. (F) ROC curves illustrate the predictive performance of Gleason score, Pathological T stages, PRDX5, and the nomogram for 5‐year BCR.

As previously mentioned, patients with high expression of PRDX5 exhibited worse BCR outcomes compared to those with low expression of PRDX5 in several datasets. Univariate Cox regression analysis revealed that PRDX5, Age, gleason score, and pathological T stage were risk factors for BCR in prostate cancer patients, with PRDX5 exhibiting the highest hazard ratio among the PRDX family (Figure 5A,B). To explore the relationship between six family members and prognosis in different stages of prostate cancer, we performed univariate cox regression analysis after classifying patients in the TCGA‐PRAD database into different stages. PRDX5 was a risk factor for BCR in prostate cancer patients with greater Gleason score, lymph node metastases, and higher pathological T stage (Figure S5, *p* < 0.05). Refer to the results above, PRDX5, Gleason score, and pathological T stage were included in a multivariate Cox regression analysis. The results demonstrated that PRDX5 (*p* = 0.038) and pathological T stage (*p* < 0.01) emerged as independent prognostic factors for prostate cancer (Figure 5C).

Given the notable prognostic significance of PRDX5 and its association with tumor staging and Gleason score in prostate cancer, we developed nomograms to predict patients' 3‐year and 5‐year BCR‐free survival outcomes. Calibration curves illustrated that these nomograms exhibited predictive performance close to the ideal model (Figure 5D). Figure 5E,F revealed that the area under the curve (AUC) of the PRDX5‐nomogram for 3‐year/5‐year BCR ranged from 0.698/0.722, 0.683/0.806, 0.864/0.884, 0.741/0.715, to 0.815/0.794 across five databases respectively. These AUC values of the nomogram surpassed those of individual predictive factors (i.e., PRDX5, pathological T stage, and Gleason score), strengthening the advantage of integrating these risk factors for prostate cancer prognosis.

### 3.6 Prognostic Value of the PRDX5‐Nomo Model

**FIGURE 6:**
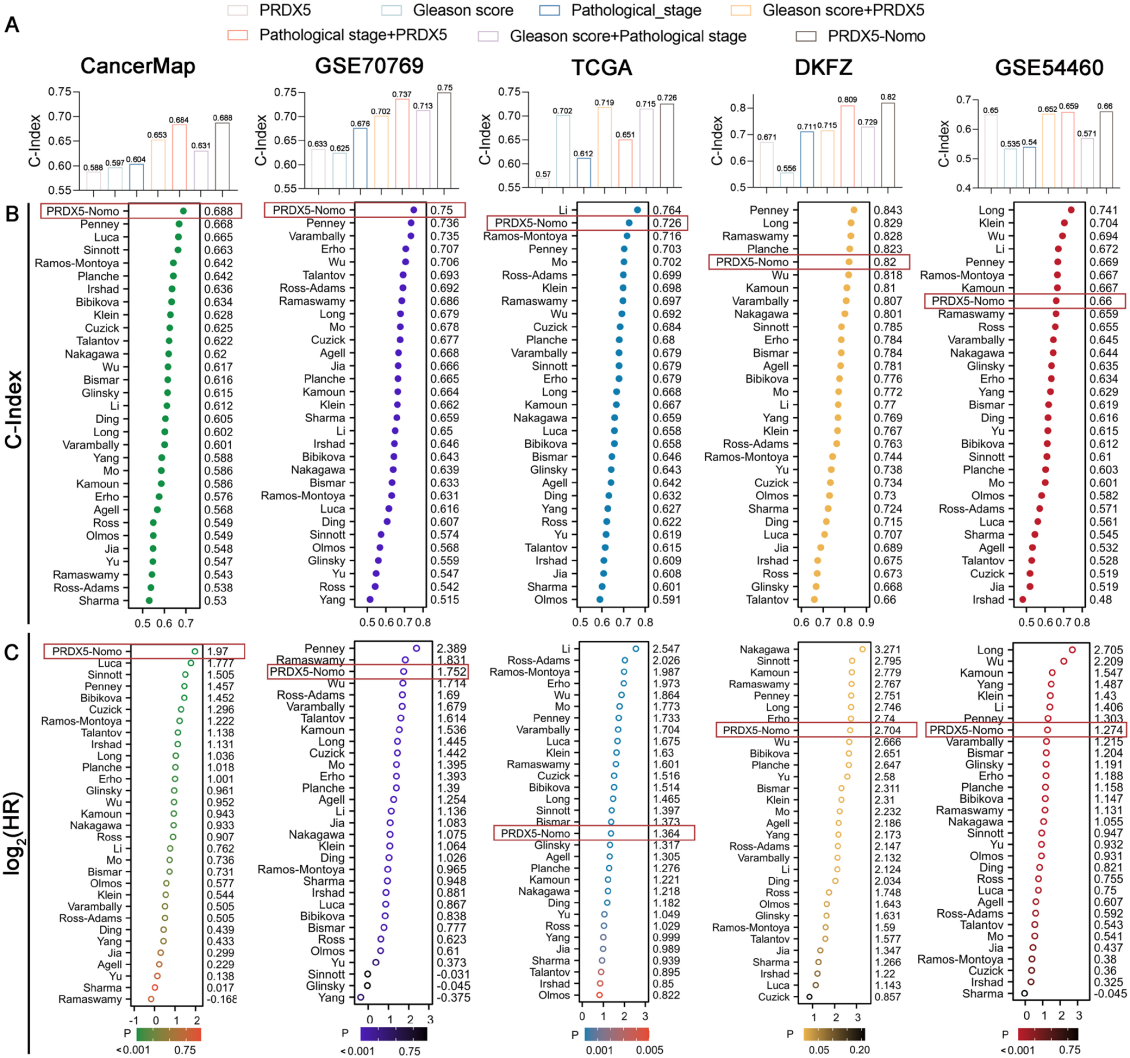
Comparison of Gene Expression‐Based Prognostic Signatures in PCa. (A) The C‐index of PRDX5, PRDX‐Nomo, Gleason score, Pathological_stage, Gleason score + PRDX5, Pathological_stage + PRDX5, and Gleason score + Pathological_stage are presented in CancerMap, GSE70769, TCGA‐PRAD, DKFZ, and GSE54460 datasets. (B) The C‐index of PRDX‐Nomo and 30 published signatures in CancerMap, GSE70769, TCGA‐PRAD, DKFZ, and GSE54460 datasets. (C) The log2(HR) of PRDX‐Nomo and 30 published signatures in CancerMap, GSE70769, TCGA‐PRAD, DKFZ, and GSE54460 datasets.

To evaluate the prognostic value of the PRDX5‐Nomo model, we calculated the C‐index of our model and PRDX5, Gleason score, Pathological_stage, Gleason score + PRDX5, Pathological_stage + PRDX5, and Gleason score + Pathological_stage in five independent cohorts. We found that the PRDX5‐Nomo model has the highest C‐index in five long‐term cohorts, with all C‐indices greater than 0.6 (Figure 6A).

We then collected the published 30 signatures associated with various biological features. The C‐index was used to estimate the probability that the predicted results will agree with the actual observed results [37] and HR was applied to represent the impact of PRDX5‐Nomo on the risk of BCR. Figure 6B illustrates the 31 signatures in each. The results showed that PRDX5‐Nomo had the highest C‐index for predicting BCR for prostate cancer in CancerMap and GSE70769, ranked second in the TCGA, fifth in the DKFZ, and eighth in the GSE54460. Both C‐indices are greater than 0.66. Our results showed that the HR of PRDX5‐Nomo ranked top 1 in CancerMap, top 3 in GSE70769, top 8 in DKFZ and GSE54460, and top 17 in TCGA. All the log2(HR) of PRDX‐Nomo were greater than 1.2, suggesting that PRDX‐Nomo may have a negative impact on BCR (Figure 6C). Last but not least, we observed that the C‐index of PRDX‐Nomo was not consistent with the ranking of HR in TCGA, which may be caused by the heterogeneity of the cohort.

### 3.7 Value of the PRDX5 in Predicting ARSI Therapy and Chemotherapy Benefits in PCa

**FIGURE 7:**
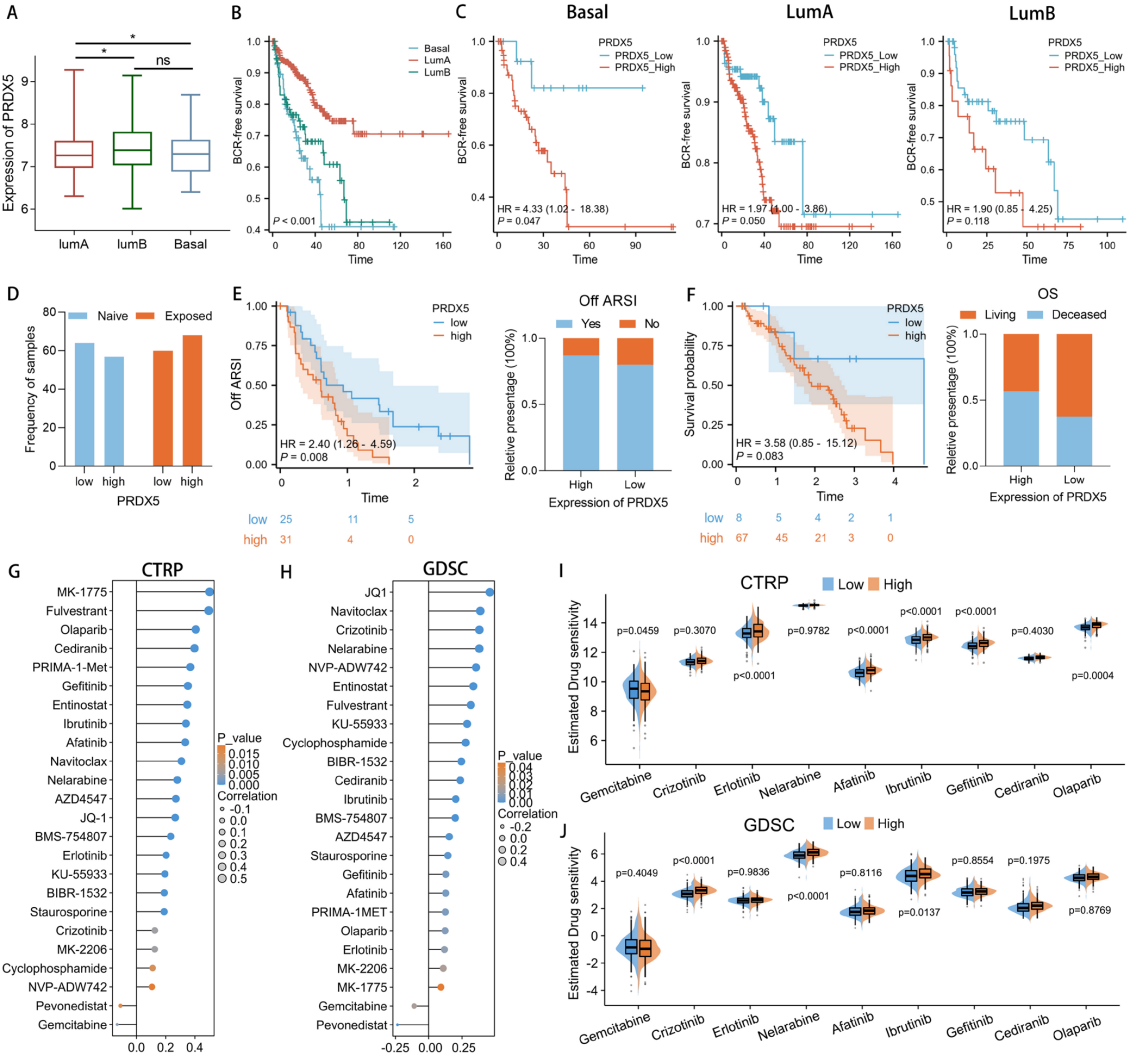
The correlation between the expression of PRDX5 and PAM50 subtypes and response to drug therapy. (A) The box plot displays the expression of PRDX5 across different PAM50 subtypes in TCGA. (B) The Kaplan–Meier curves depict the BCR status of PCa patients in TCGA, stratified by Luminal A, Luminal B, and Basal subtypes. (C) The diagrams display the BCR free survival for PCa patients of two PRDX5 subgroups within each PAM50 subtype. (D) The PRDX5 expression constitution of PCa patients before or after ARSI therapy. (E) The Kaplan–Meier curve of off ARSI time (*p* = 0.008), and the off ARSI constitution of PCa patients in the two PRDX5 subgroups. (F) The Kaplan–Meier curve of overall survival time (*p* = 0.083) and outcome constitution (living or deceased) of PCa patients in the two PRDX5 subgroups. (G, H) The lollipop plot illustrates the correlation between multiple drug sensitivities and PRDX5 expression levels in the CTRP (G) and GDSC (H) databases. (I, J) The grouped box plot illustrates the drug sensitivity of nine clinical drugs in two PRDX5 subgroups in the CTRP (I) and GDSC (J) databases.

The PAM50 algorithm is able to differentiate between luminal‐ and basal‐like subtypes in prostate cancer. These subtypes exhibit distinct clinical characteristics and show varying responses to postoperative androgen deprivation therapy (ADT) [38, 39]. The initial step is to classify the samples from the TCGA dataset into luminal A (LumA), luminal B (LumB) and basal phenotypes. The expression of PRDX5 across different PAM50 subtypes is illustrated in Figure 7A. The expression in LumB and basal phenotypes was higher than that in the LumA phenotype (*p* < 0.05). Significant shortening in BCR‐free survival time among patients in LumB and basal phenotypes compared to that in LumA subtypes is shown (Figure 7B). We further constructed BCR‐free survival curves for patients within each of the three PAM50 subtypes, respectively. Notably, patients with higher PRDX5 expression in both the basal and luminal A subtypes exhibited a higher propensity for BCR. Similarly, this trend was evident within the luminal B subtype, though there was a lack of statistical support (Figure 7C).

The next‐generation ARSIs, especially enzalutamide and abiraterone acetate, were identified to perform significant advancement in PCa therapy. However, both histology, genomics, and transcriptomics factors may be associated with the failure of treatment and lead to disease progression [40, 41, 42, 43]. Novel prognostic markers in metastatic castration‐resistant prostate cancer were urgently needed. We explored the value of the PRDX5 in predicting patient response to ARSI within an independent ARSI cohort [44]. As shown in Figure 7D, the frequency of patients in the high PRDX5 group was higher after exposing them to ARSI therapy, and there was a reverse trend in patients who did not receive treatment. Patients with low PRDX5 expression levels had a longer duration of ARSI therapy compared to those with high PRDX5 expression levels. The proportion of patients who remained on ARSI treatment was lower in the high PRDX5 expression level group than in the low PRDX5 expression level group (Figure 7E). Notably, patients with low PRDX5 expression levels exhibited a more favorable outcome in terms of living compared to those with high PRDX5 expression. The high PRDX5 expression level group showed a significantly higher proportion of deceased patients compared to the low PRDX5 expression level group (Figure 7F).

So as to evaluating PRDX5's predictive capacity for drug sensitivity, we conducted analyses using the CTRP and the GDSC datasets (Figure 7G–J). Intriguingly, PRDX5 exhibited a positive correlation with sensitivity to MK‐1775, MK‐2206, Erlotinib, Olaparib, Afatinib, Gefitinib, Staurosporine, AZD4547, BMS‐754807, Ibrutinib, Cediranib, BIBR‐1532, Cyclophosphamide, KU‐55933, Fulvestrant, Entinostat, NVP‐ADW742, Nelarabine, Crizotinib and Navitoclax in both the CTRP and GDSC databases. Furthermore, PRDX5 exhibited a negative correlation with sensitivity to Pevonedistat, Gemcitabine. In summary, our data indicate that PRDX5 may have a significant influence on the clinical use of ARSI or chemotherapeutic medications.

### 3.8 Immunohistochemistry Assay Confirmed a Positive Correlation Between PRDX5 Expression and Malignant Progression in PCa

**FIGURE 8:**
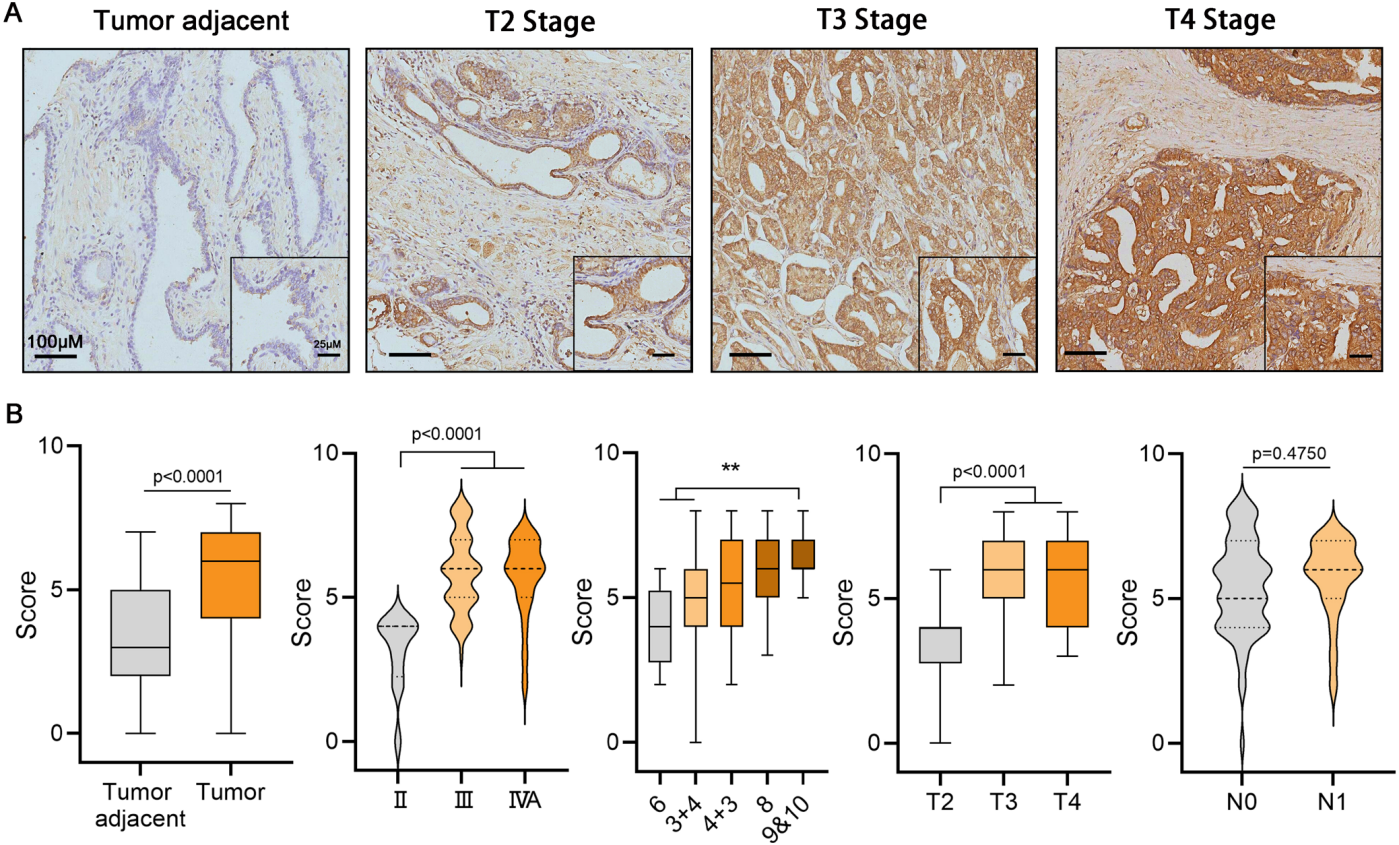
Representative IHC staining for PRDX5 in PCa tumor‐adjacent or tumor tissue in a Tissue microarrays (A) and the staining intensity score was counted and compared (B) (*p < 0.05, **p < 0.01).

IHC was utilized to validate the expression of PRDX5 in PCa tissues. The staining intensity of PRDX5 in PCa tissues was significantly higher compared to adjacent non‐cancerous tissues (Figure 8A,B, *p* < 0.0001). Moreover, the staining intensity was notably elevated in AJCC stage III and stage IVa compared to stage II (*p* < 0.0001). Additionally, tissues with Gleason scores of 9&10 exhibited stronger staining intensity compared to those with Gleason scores of 6 and 3 + 4 (*p* < 0.01). The staining intensity in Gleason score of 4 + 3 or 8 was higher than that in the score of 3 + 4 or 6, though there was no statistical significance. Furthermore, the staining intensity in pathological T stages T3 and T4 was significantly higher than in T2 (*p* < 0.0001). Although not statistically significant, a trend was observed where the staining intensity in lymph node metastasis stage N1 was higher than in N0.

### 3.9 Biological Mechanisms Prediction of PRDX5 in PCa

**FIGURE 9:**
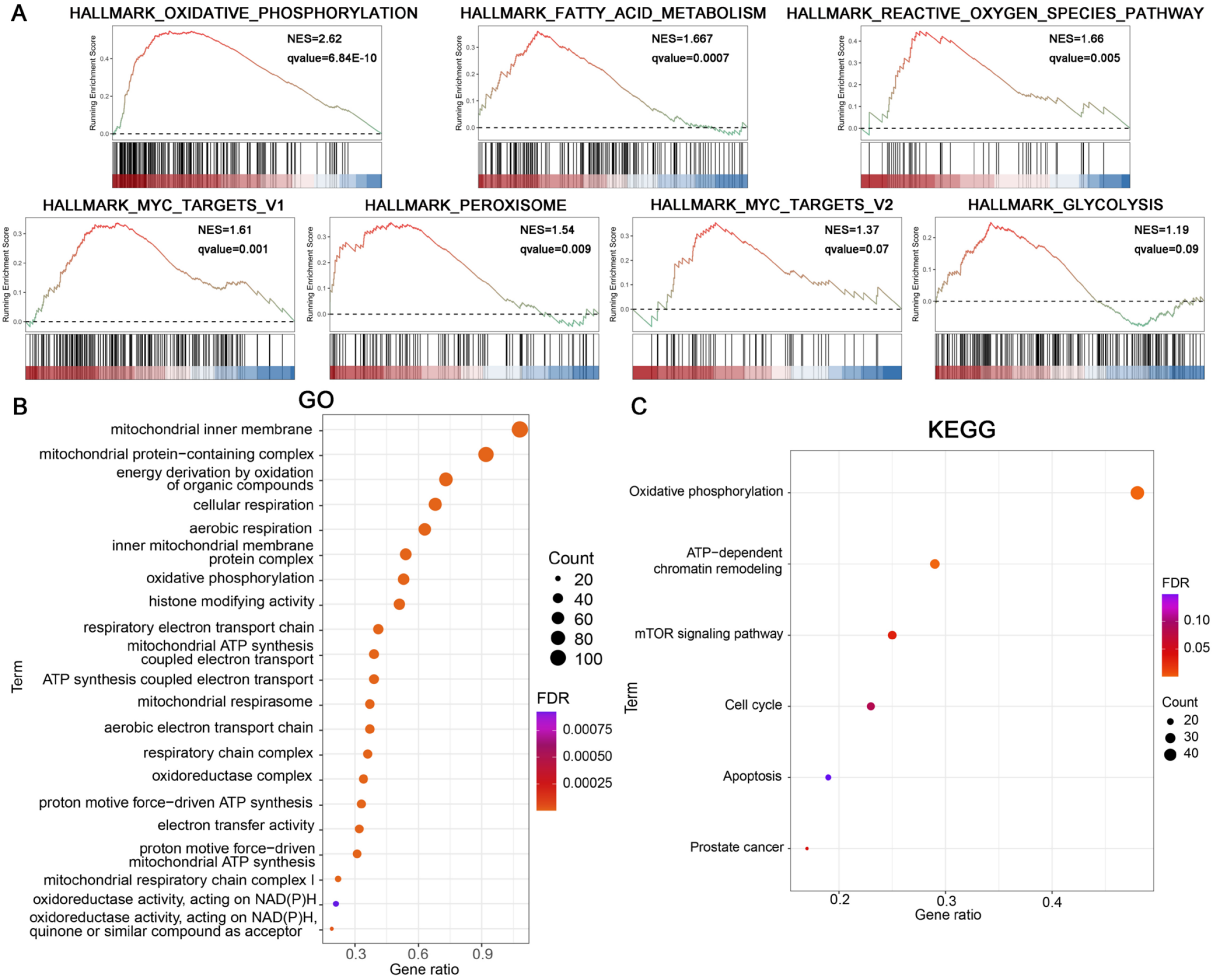
Biological mechanisms enrichment analysis of PRDX5 in PCa. (A) GSEA plots display functional pathways enriched in differentially expressed genes (DEGs). (B, C) The bubble plots display GO and KEGG pathway enrichment data for related genes (RGs).

Following the high/low PRDX5 expression level groups were generated in the TCGA‐PRAD dataset, the differential expression analysis between groups was performed. GSEA results indicated that the DEGs in the high PRDX5 expression level group were primarily enriched in Oxidative Phosphorylation (NES = 2.62, *q* < 0.001), Fatty Acid Metabolism (NES = 1.667, *q* < 0.001), ROS Pathway (NES = 1.66, qvalue = 0.005), Myc Targets V1 (NES = 1.61, qvalue = 0.001), Peroxisome (NES = 1.54, qvalue = 0.009), Myc Targets V2 (NES = 1.37, qvalue = 0.07), Glycolysis (NES = 1.19, qvalue = 0.09) (Figure 9A). GO and KEGG pathway enrichment analysis revealed that the PRDX5 related genes (RGs) were mainly involved in Mitochondria‐related functions, Cellular Respiration, and Oxidative phosphorylation (Figure 9B,C).

### 3.10 Depletion of PRDX5 Induces Apoptosis and Affects the Sensitivity of ARSI Therapy via ROS Accumulation

**FIGURE 10:**
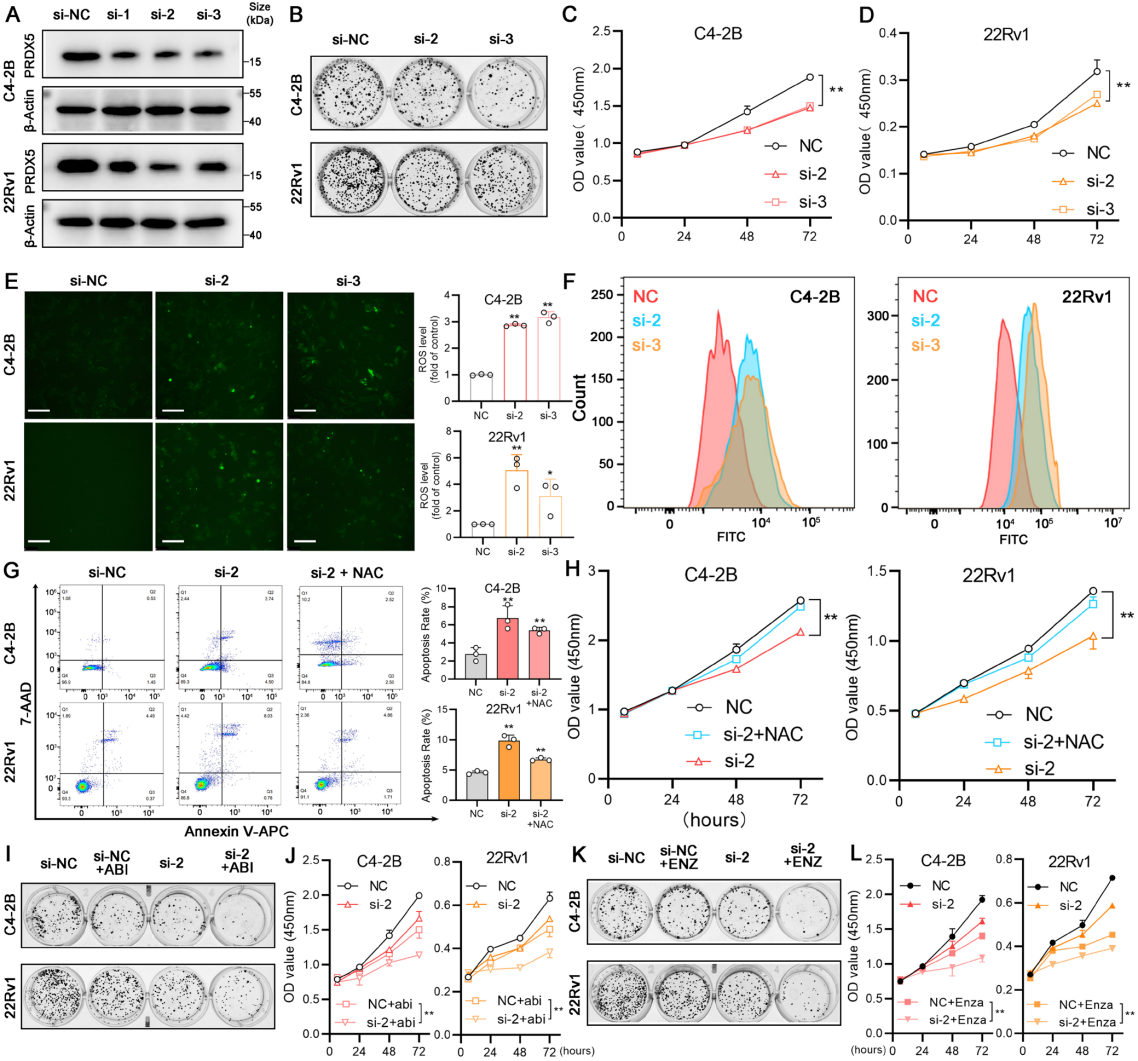
Depletion of PRDX5 induces apoptosis and affects the sensitivity of ARSI therapy via ROS accumulation. (A) Verification of PRDX5 down‐regulated C4‐2B and 22Rv1cell lines. (B) Colony formation of PRDX5 down‐regulated C4‐2B and 22Rv1cell lines compared with the negative control (si‐NC) group. (C) CCK‐8 assays of PRDX5 down‐regulated C4‐2B cell lines compared with si‐NC group. (D) CCK‐8 assays of PRDX5 down‐regulated 22Rv1 cell lines compared with the si‐NC group. E. Images of cellular ROS levels were captured (scale bar: 250 μm). (F) The ROS levels of 22Rv1 and C4‐2B cells were analyzed by flow cytometry. (G) The apoptosis flow cytometry assays of C4‐2B and 22Rv1 cell lines. (H) Combined application of si‐PRDX5 and NAC was measured by using the CCK‐8 assay. (I) Colony formation of PRDX5 down‐regulated C4‐2B and 22Rv1cell lines comparing with si‐NC under Abiraterone treatment. (J) CCK‐8 assays of PRDX5 down‐regulated C4‐2B and 22Rv1 cell lines compared with si‐NC under Abiraterone treatment. (K) Colony formation of PRDX5 down‐regulated C4‐2B and 22Rv1cell lines compared with si‐NC under Enzalutamide treatment. (L) CCK‐8 assays of PRDX5 down‐regulated C4‐2B and 22Rv1 cell lines comparing with si‐NC under Enzalutamide treatment. (*p < 0.05, **p < 0.01, Abi: Abiraterone, Enz: Enzalutamide).

PRDX5 downregulation in 22Rv1 and C4‐2B cell lines was successfully achieved and verified by western blotting (Figure 10A, *p* < 0.05). The second siRNA showed better protein depletion efficacy in both cell lines and followed by the third siRNA (Figure S6B, *p* < 0.05). We found that downregulating PRDX5 reduced the colony formation ability of both 22Rv1 and C4‐2B cells (Figure 10B, Figure S6B, *p* < 0.05). Moreover, when PRDX5 was downregulated, the growth curves of 22Rv1 and C4‐2B cells were inhibited (Figure 10C,D, *p* < 0.05). As the main function of PRDX5 is to remove excess ROS to maintain the relative stability of the redox state in the cell, we measured ROS levels of two cell lines after interfering with the PRDX5 expression. As shown in Figure 10E, green fluorescence represents the DCFH‐DA staining, and we found that the intracellular ROS production enhanced significantly. Similar results can be obtained from flow cytometry experiments (Figure 10F, Figure S6C, *p* < 0.05). Next, the annexin V‐APC/7‐AAD double‐staining assay showed that the depletion of PRDX5 induced apoptosis in 22Rv1 and C4‐2B cells. Meanwhile, these apoptosis alterations were significantly reversed by pretreatment with N‐acetylcysteine (NAC), a ROS scavenger (Figure 10G). CCK‐8 assays revealed a similar result in the two cell lines (Figure 10H, *p* < 0.05). Taken together, these results showed that the enhanced ROS levels contribute to the cytotoxicity of PRDX5 depletion in two CRPC cell lines. Moreover, the expression of PRDX5 in CRPC cells also affects the sensitivity to the ARSI therapy. As shown in Figure 10I,J, cells with lower PRDX5 levels exhibited higher sensitivity to abiraterone therapy (Figure S6D, *p* < 0.05). Similarly, depletion of PRDX5 also enhanced the sensitivity to enzalutamide therapy in 22Rv1 and C4‐2B (Figure S6E, *p* < 0.05), consistent with the result in Figure 7.

## 4 Discussion

The current primary methods for clinically assessing the aggressiveness of prostate cancer include TNM staging, digital rectal examination (DRE), repeated prostate tissue needle biopsies to determine the Gleason score, and serum PSA testing [45]. However, large amounts of patients still face overtreatment or undertreatment due to insufficient predictive accuracy even after considering all these indicators [46, 47]. Oxidative stress is a hallmark of cancer. Tumorigenesis and progression have been correlated with elevated amounts of ROS and a corresponding upregulation of antioxidant expression. Among the most crucial antioxidants are PRDXs, which are widely distributed across various types of cancer. In this study, we investigated the value of the PRDXs family in the occurrence, progression, prognosis, and treatment response of PCa and predict the potential function of the key character, PRDX5, may participate.

The level of DNA CNAs across the genome, which refers to the fraction of the tumor genome impacted by CNAs, was found to be linked with BCR and post‐surgery metastasis in PCa [48]. We found that the PRDXs family exhibits various extents of CNAs in PCa across multiple public databases, with PRDX5 showing the highest frequency of amplification within. The expression analysis in the TCGA transcriptome aligns with this finding, showing that PRDX5 was significantly upregulated in tumors compared to normal tissues, with higher expression levels observed in samples with advanced clinical grades and higher Gleason Score. Combining single‐cell transcriptome and IHC both from online and in tissue microarray results, it was discovered that cells exhibiting elevated levels of PRDX5 expression are mainly localized in epithelial cells. This indicates that the raised expression of PRDX5 in prostate cancer epithelial cells plays a pivotal role in the advancement of tumors. This finding aligns with previous observations regarding the antioxidant function of PRDXs family members in tumor cells.

Meanwhile, by integrating results from multiple databases, we found that different PRDXs family members have varying impacts on the prognosis of PCa, with PRDX5 playing the most significant role. Furthermore, PRDX5 has been recognized as a distinct predictive risk factor for PCa. These findings indicated that PRDX5 may have a more prominent involvement in the development of prostate cancer compared to other members of its family. Prior research has observed increased expression levels of PRDX1 in numerous cancer types, including non‐small cell lung cancer (NSCLC), ovarian cancer (OC), prostate cancer, colorectal cancer, and gastric cancer [49, 50, 51, 52]. Oral squamous cell carcinoma was indicated to have overexpression of PRDX1, PRDX2, and PRDX6 [53, 54, 55, 56]. Studies have demonstrated that downregulation of PRDX2 inhibits tumor cell proliferation, migration, and invasion in gastric cancer and colorectal cancer [57, 58]. Moreover, PRDX2 has been shown to promote the progression of prostate cancer by activating the androgen receptor (AR) signaling pathway [59]. Regarding PRDX3, research suggested its association with metastasis and poor survival rates in uveal melanoma [60, 61], and silencing PRDX3 has been found to inhibit liver cancer cell growth and promote invasion [51]. Furthermore, the tumorigenic role of PRDX4 has been confirmed in lung cancer, leukemia, and glioblastoma [20, 62, 63]. PRDX6, the only non‐selenocysteine glutathione peroxidase in the PRDXs family, has been implicated in promoting late‐stage G2/M arrest in liver cancer cells, inhibiting tumor proliferation in hepatocarcinoma [64]. Additionally, PRDX6 knockout has been shown to suppress the malignant progression of intrahepatic cholangiocarcinoma [65]. On the basis of these past studies, our research innovatively compared the prognostic impact of all six family members in prostate cancer and found that PRDX5 has the strongest predictive value for poor prognosis in PCa patients and is most closely associated with adverse outcomes. After validation by in vitro experiments, we found that depletion of PRDX5 induces apoptosis and regulates the sensitivity of ARSI therapy via ROS accumulation.

PRDX5 is an atypical 2‐Cys antioxidant with broad substrate specificity, capable of counteracting endogenous and exogenous peroxides, including H_2_O_2_, alkyl hydroperoxides, and peroxynitrite [66, 67]. It is widely distributed in cells. Since mitochondria and peroxisomes are the main sources of ROS/reactive nitrogen species within cells, recent studies have highlighted the upregulation of PRDX5 in various tumor tissues, enhancing tumorigenic phenotypes and metastatic potential [68]. Studies have shown that PRDX5 protects gastric cancer cells from apoptosis by reducing intracellular ROS accumulation, thus promoting their proliferation and invasiveness [69, 70]. In xenograft mouse models, PRDX5 overexpression has been shown to promote tumor growth of colorectal cancer cells [71]. Moreover, PRDX5 has been found to play a tumor‐promoting role in thyroid cancer [72]. Research on PRDX5 in PCa is limited, and its mechanisms in this context are not well understood. Previous work has demonstrated that the expression of PRDX5 in PC‐3 cells, a type of human prostate cancer, notably rises when exposed to oxidative stress [73]. Recent studies have shown that PRDX5 promotes resistance to AR inhibitors and the development of castration‐resistant prostate cancer (CRPC). Specifically, inhibiting PRDX5 can suppress DTP cell proliferation in culture, hinder CRPC progression in animal models, and stabilize PSA progression and metastatic lesions in patients, indicating that PRDX5 could be a therapeutic target for treatment‐resistant prostate cancer [74]. This aligns with our findings on promoting prostate cancer progression and regulating the ARSI therapy response.

GSEA and pathway enrichment analysis indicated that high PRDX5 expression is often accompanied by the enrichment of certain pathways, including mitochondria‐related functions, cellular respiration, fatty acid metabolism, ROS pathway, Myc Targets, peroxisome, and glycolysis. Elevated ROS levels have been shown to trigger apoptosis through various downstream pathways, with mitochondrial dysfunction being the most critical [75]. On the contrary, energy metabolism processes related to oxidative phosphorylation and the tricarboxylic acid (TCA) cycle will be altered. The TCA cycle provides intermediates for the production of lipids, proteins, and nucleotides [76]. ROS mainly mediates signal processes, crosstalk with metabolism, tumor microenvironment, and immune functions in cancer [77]. Integrating with the bioinformatics analysis, high expression of PRDX5 may enhance energy supply for tumor cells, thereby promoting cell cycle progression, which indicates that the metabolism impact of ROS or PRDXs family in PCa may merit further investigation.

In an effort to conduct a more thorough examination of the medical importance of PRDX5, a nomogram was created using PRDX5 and clinical characteristics. The calibration curves for 3‐year and 5‐year survival predictions closely approximated the ideal values. The AUC values for the 3‐year and 5‐year nomograms were greater than the AUC values for each individual clinical predictor. Therefore, our novel nomogram can provide clinical assistance based on the individual characteristics of PCa patients. Comparing with published 30 signatures associated with various biological features, the PRDX5‐Nomo model showed good predictive power for BCR in clinical patients. In addition, our model merely needs one expression of gene, which is easier to assist clinicians than the rest of the polygenic models. Except for predicting prognosis, PRDX5 can also distinguish molecular subtypes and predict drug response in PCa. Notable among primary tumor classification systems is the predictive analysis of microarrays 50 (PAM50) gene panel developed from large‐scale transcriptome assessments of breast cancer [78]. Given that prostate and breast cancer are both hormonally driven tumors and share many oncogenic pathways, PAM50 was proved to identify luminal and basal‐like subtypes in prostate cancer and these subtypes could differ in clinical outcomes and treatment response [38]. Additionally, PRDX5 exhibited a negative correlation with sensitivity to Pevonedistat, Gemcitabine, suggesting that it could be a novel option for PCa patients with high levels of PRDX5.

## 5 Conclusion

In conclusion, this study offers new evidence for determining that the expression of PRDX5 is associated with advanced tumor grade, poor prognosis, and the suboptimal response to multiple therapies in PCa within the PRDXs family. The expression of PRDX5 and the Gleason score and pathologic T stage of PCa patients were included to construct a nomogram to improve the current BCR prediction clinically. Combining the bioinformatics and in vitro experiment results, PRDX5 induces apoptosis and affects the sensitivity of ARSI therapy via ROS accumulation in CRPC. Last but not least, our study provides new insights into precision medicine in PCa and provides a reference for further research on PRDX5.

## Author Contributions

**Shan Tang:** funding acquisition, project administration, conceptualization. **Jinchuang Li:** data curation, formal analysis. **Weicheng Tian:** formal analysis. **Yuanfa Feng:** validation. **Yulin Deng:** validation. **Zeheng Tan:** validation. **Zhaodong Han:** data curation, visualization. **Huichan He:** investigation, visualization, validation, data curation. **Yongding Wu:** data curation, visualization. **Chuyang Huang:** investigation, resources. **Keping Ning:** investigation, resources. **Feng Liu:** data curation, visualization. **Hongwei Luo:** writing – original draft, funding acquisition, methodology. **Shanghua Cai:** writing – review and editing, formal analysis. **Jianheng Ye:** writing – original draft, funding acquisition, methodology. **Weide Zhong:** writing – review and editing, funding acquisition, conceptualization, supervision.

## Consent

The authors have nothing to report.

## Conflicts of Interest

The authors declare no conflicts of interest.

## Supporting information


**Figure S1.** Bubble heatmap showing marker genes of eight major cell types in GSE141445 and GSE157703 dataset. Dot size indicates fraction of expressing cells, and represents to expression levels.

**Figure S2.** Depicting the expression of rest 5 genes in single‐cell sequencing with uniform manifold approximation and projection (UMAP) using GSE141445 and GSE157703 database.

**Figure S3.** Immunohistochemistry staining of the rest 5 genes in Human protein altas database.

**Figure S4.** Kaplan–Meier curves illustrate the BCR status of prostate cancer patients across Cambridge (A) and GSE116918 (B) database.

**Figure S5.** (A) Univariate analyses of PRDXs in prostate cancer patients with Gleason score ≥ 7 from TCGA. (B) Univariate analyses of PRDXs in prostate cancer patients with clinical N1 stage from TCGA. (C) Univariate analyses of PRDXs in prostate cancer patients with clinical N0 stage from TCGA. (D) Univariate analyses of PRDXs in prostate cancer patients with pathological T stage ≥ 3 from TCGA. (E) Univariate analyses of PRDXs in prostate cancer patients with pathological T stage < 3 from TCGA.

**Figure S6.** (A) Protein quantification assay for western blot experiment in Figure 10A. (B) Statistics analysis of colony formation assay in Figure 10B. (C) Statistics analysis of ROS flow cytometry assay in Figure 10F. (D) Statistics analysis of colony formation assay in Figure 10I. (E) Statistics analysis of colony formation assay in Figure 10K (*p < 0.05, **p < 0.01).

## Data Availability

RNA‐sequencing data from 100 normal prostate tissue samples were retrieved from the Genotype‐Tissue Expression (GTEx, https://gtexportal.org/). Seven independent public datasets (including TCGA, CancerMap (GSE94767), DKFZ, GSE54460, GSE70769, Cambridge (GSE70768) and GSE116918) were accessed from The Cancer Genome Atlas (TCGA, http://portal.gdc.cancer.gov/) and Gene Expression Omnibus (GEO, https://www.ncbi.nlm.nih.gov/geo/). Single‐cell sequencing analysis data were obtained from the sequencing results of GSE157703 and GSE141445 in GEO. Information on 545 drugs from the Cancer Therapeutics Response Portal (CTRP) database and 198 drugs from the Genomics of Drug Sensitivity in Cancer (GDSC) database was retrieved. Furthermore, data pertaining to androgen receptor signaling inhibitor (ARSI) therapy cohorts were obtained from Github (https://github.com/cBioPortal/datahub/tree/master/public/prad_su2c_2019). To further validate the predictive performance of PRDX5‐Nomo signature, the C‐indexes and hazard ratios (HR) of 30 gene expression prognostic signatures trained with the robust Cox‐Ridge algorithm were downloaded from the PCaDB database (http://bioinfo.jialab‐ucr.org/PCaDB/).

## References

[1] R. L. Siegel , A. N. Giaquinto , and A. Jemal , “Cancer Statistics, 2024,” CA: A Cancer Journal for Clinicians 74 (2024): 12–49. 38230766 10.3322/caac.21820

[2] F. Bray , M. Laversanne , H. Sung , et al., “Global Cancer Statistics 2022: GLOBOCAN Estimates of Incidence and Mortality Worldwide for 36 Cancers in 185 Countries,” CA: A Cancer Journal for Clinicians 74, no. 3 (2024): 229–263, 10.3322/caac.21834. 38572751

[3] T. Van den Broeck , R. C. N. van den Bergh , N. Arfi , et al., “Prognostic Value of Biochemical Recurrence Following Treatment With Curative Intent for Prostate Cancer: A Systematic Review,” European Urology 75 (2019): 967–987. 30342843 10.1016/j.eururo.2018.10.011

[4] S. J. Freedland , E. B. Humphreys , L. A. Mangold , et al., “Death in Patients With Recurrent Prostate Cancer After Radical Prostatectomy: Prostate‐Specific Antigen Doubling Time Subgroups and Their Associated Contributions to All‐Cause Mortality,” Journal of Clinical Oncology: Official Journal of the American Society of Clinical Oncology 25 (2007): 1765–1771. 17470867 10.1200/JCO.2006.08.0572

[5] R. J. Rebello , C. Oing , K. E. Knudsen , et al., “Prostate Cancer,” Nature Reviews Disease Primers 7 (2021): 9. 10.1038/s41572-020-00243-0 33542230

[6] S. J. Freedland , E. B. Humphreys , L. A. Mangold , et al., “Risk of Prostate Cancer‐Specific Mortality Following Biochemical Recurrence After Radical Prostatectomy,” Jama 294 (2005): 433–439. 16046649 10.1001/jama.294.4.433

[7] E. C. Cheung and K. H. Vousden , “The Role of ROS in Tumour Development and Progression,” Nature Reviews. Cancer 22 (2022): 280–297. 35102280 10.1038/s41568-021-00435-0

[8] Y. Wang , H. Qi , Y. Liu , et al., “The Double‐Edged Roles of ROS in Cancer Prevention and Therapy,” Theranostics 11 (2021): 4839–4857. 33754031 10.7150/thno.56747 PMC7978298

[9] C. Fan , X. Yang , L. Yan , and Z. Shi , “Oxidative Stress Is Two‐Sided in the Treatment of Acute Myeloid Leukemia,” Cancer Medicine 13 (2024): e6806. 38715546 10.1002/cam4.6806 PMC11077289

[10] D. Trachootham , J. Alexandre , and P. Huang , “Targeting Cancer Cells by ROS‐Mediated Mechanisms: A Radical Therapeutic Approach?,” Nature Reviews. Drug Discovery 8 (2009): 579–591. 19478820 10.1038/nrd2803

[11] Z. Zou , H. Chang , H. Li , and S. Wang , “Induction of Reactive Oxygen Species: An Emerging Approach for Cancer Therapy,” Apoptosis: An International Journal on Programmed Cell Death 22, no. 11 (2017): 1321–1335, 10.1007/s10495-017-1424-9. 28936716

[12] T. Maser , M. Rich , D. Hayes , et al., “Tolcapone Induces Oxidative Stress Leading to Apoptosis and Inhibition of Tumor Growth in Neuroblastoma,” Cancer Medicine 6 (2017): 1341–1352. 28429453 10.1002/cam4.1065 PMC5463066

[13] S. Castelli , F. Ciccarone , P. De Falco , and M. R. Ciriolo , “Adaptive Antioxidant Response to Mitochondrial Fatty Acid Oxidation Determines the Proliferative Outcome of Cancer Cells,” Cancer Letters 554 (2023): 216010, 10.1016/j.canlet.2022.216010. 36402229

[14] X. Xu , C. Wang , P. Zhang , et al., “Enhanced Intracellular Reactive Oxygen Species by Photodynamic Therapy Effectively Promotes Chemoresistant Cell Death,” International Journal of Biological Sciences 18, no. 1 (2022): 374–385, 10.7150/ijbs.66602. 34975339 PMC8692137

[15] Q. Cui , J.‐Q. Wang , Y. G. Assaraf , et al., “Modulating ROS to Overcome Multidrug Resistance in Cancer,” Drug Resistance Updates 41 (2018): 1–25. 30471641 10.1016/j.drup.2018.11.001

[16] Y. Li , G. Jin , N. Liu , H. Guo , and F. Xu , “The Post‐Chemotherapy Changes of Tumor Physical Microenvironment: Targeting Extracellular Matrix to Address Chemoresistance,” Cancer Letters 582 (2024): 216583, 10.1016/j.canlet.2023.216583. 38072368

[17] S. G. Rhee , “Overview on Peroxiredoxin,” Molecules and Cells 39 (2016): 1–5. 26831451 10.14348/molcells.2016.2368 PMC4749868

[18] Y. Kim and H. H. Jang , “The Role of Peroxiredoxin Family in Cancer Signaling,” Journal of Cancer Prevention 24 (2019): 65–71. 31360686 10.15430/JCP.2019.24.2.65 PMC6619859

[19] Y. J. Lee , “Knockout Mouse Models for Peroxiredoxins,” Antioxidants 9 (2020): 182. 32098329 10.3390/antiox9020182 PMC7070531

[20] K. K. Palande , R. Beekman , L. E. van der Meeren , H. B. Beverloo , P. J. M. Valk , and I. P. Touw , “The Antioxidant Protein Peroxiredoxin 4 Is Epigenetically Down Regulated in Acute Promyelocytic Leukemia,” PLoS One 6 (2011): e16340. 21283726 10.1371/journal.pone.0016340 PMC3024432

[21] B. Zhang , K. Wang , G. He , et al., “Polymorphisms of Peroxiredoxin 1, 2 and 6 Are Not Associated With Esophageal Cancer,” Journal of Cancer Research and Clinical Oncology 138 (2012): 621–626. 22215146 10.1007/s00432-011-1119-5 PMC11824428

[22] H. C. Whitaker , D. Patel , W. J. Howat , et al., “Peroxiredoxin‐3 Is Overexpressed in Prostate Cancer and Promotes Cancer Cell Survival by Protecting Cells From Oxidative Stress,” British Journal of Cancer 109 (2013): 983–993. 23880827 10.1038/bjc.2013.396 PMC3749568

[23] S. G. Rhee , H. Z. Chae , and K. Kim , “Peroxiredoxins: A Historical Overview and Speculative Preview of Novel Mechanisms and Emerging Concepts in Cell Signaling,” Free Radical Biology & Medicine 38 (2005): 1543–1552. 15917183 10.1016/j.freeradbiomed.2005.02.026

[24] X. Guo , H. Noguchi , N. Ishii , et al., “The Association of Peroxiredoxin 4 With the Initiation and Progression of Hepatocellular Carcinoma,” Antioxidants & Redox Signaling 30 (2019): 1271–1284. 29687726 10.1089/ars.2017.7426

[25] M. Bajor , A. Graczyk‐Jarzynka , K. Marhelava , et al., “Triple Combination of Ascorbate, Menadione and the Inhibition of Peroxiredoxin‐1 Produces Synergistic Cytotoxic Effects in Triple‐Negative Breast Cancer Cells,” Antioxidants 9 (2020): 320. 32316111 10.3390/antiox9040320 PMC7222372

[26] H. Yan , X. Cai , S. Fu , X. Zhang , and J. Zhang , “PRDX3 Promotes Resistance to Cisplatin in Gastric Cancer Cells,” Journal of Cancer Research and Therapeutics 18 (2022): 1994–2000. 36647961 10.4103/jcrt.jcrt_970_22

[27] C. Song , G. Xiong , S. Yang , et al., “PRDX1 Stimulates Non‐Small‐Cell Lung Carcinoma to Proliferate via the Wnt/β‐Catenin Signaling,” Panminerva Medica 65 (2023): 37–42. 32881473 10.23736/S0031-0808.20.03978-6

[28] M. B. Hampton , K. A. Vick , J. J. Skoko , and C. A. Neumann , “Peroxiredoxin Involvement in the Initiation and Progression of Human Cancer,” Antioxidants & Redox Signaling 28 (2018): 591–608. 29237274 10.1089/ars.2017.7422 PMC9836708

[29] T. E. Forshaw , R. Holmila , K. J. Nelson , et al., “Peroxiredoxins in Cancer and Response to Radiation Therapies,” Antioxidants 8 (2019): 11. 30609657 10.3390/antiox8010011 PMC6356878

[30] R. Li , J. Zhu , W.‐D. Zhong , and Z. Jia , “Comprehensive Evaluation of Machine Learning Models and Gene Expression Signatures for Prostate Cancer Prognosis Using Large Population Cohorts,” Cancer Research 82 (2022): 1832–1843. 35358302 10.1158/0008-5472.CAN-21-3074

[31] Y. Zhou , C. Lin , L. Zhu , R. Zhang , L. Cheng , and Y. Chang , “Nomograms and Scoring System for Forecasting Overall and Cancer‐Specific Survival of Patients With Prostate Cancer,” Cancer Medicine 12 (2023): 2600–2613. 35993499 10.1002/cam4.5137 PMC9939188

[32] J. S. Parker , M. Mullins , M. C. U. Cheang , et al., “Supervised Risk Predictor of Breast Cancer Based on Intrinsic Subtypes,” Journal of Clinical Oncology: Official Journal of the American Society of Clinical Oncology 41 (2023): 4192–4199. 37672882 10.1200/JCO.22.02511

[33] S. Cai , Y. Feng , J. Ye , et al., “The Prognostic Roles of CYP19A1 Expression in Bladder Cancer Patients of Different Genders,” Translational Andrology and Urology 10 (2021): 3579–3590. 34733654 10.21037/tau-21-400 PMC8511542

[34] J. Ye , S. Cai , Y. Feng , et al., “Metformin Escape in Prostate Cancer by Activating the PTGR1 Transcriptional Program Through a Novel Super‐Enhancer,” Signal Transduction and Targeted Therapy 8, no. 1 (2023): 303, 10.1038/s41392-023-01516-2. 37582751 PMC10427640

[35] B. S. Taylor , N. Schultz , H. Hieronymus , et al., “Integrative Genomic Profiling of Human Prostate Cancer,” Cancer Cell 18, no. 1 (2010): 11–22, 10.1016/j.ccr.2010.05.026. 20579941 PMC3198787

[36] H. H. Cheng , A. O. Sokolova , E. M. Schaeffer , E. J. Small , and C. S. Higano , “Germline and Somatic Mutations in Prostate Cancer for the Clinician,” Journal of the National Comprehensive Cancer Network 17, no. 5 (2019): 515–521, 10.6004/jnccn.2019.7307. 31085765

[37] M. Herrmann , P. Probst , R. Hornung , V. Jurinovic , and A.‐L. Boulesteix , “Large‐Scale Benchmark Study of Survival Prediction Methods Using Multi‐Omics Data,” Briefings in Bioinformatics 22, no. 3 (2021): bbaa167, 10.1093/bib/bbaa167. 32823283 PMC8138887

[38] S. G. Zhao , S. L. Chang , N. Erho , et al., “Associations of Luminal and Basal Subtyping of Prostate Cancer With Prognosis and Response to Androgen Deprivation Therapy,” JAMA Oncology 3 (2017): 1663–1672. 28494073 10.1001/jamaoncol.2017.0751 PMC5824281

[39] I. M. Coleman , N. DeSarkar , C. Morrissey , et al., “Therapeutic Implications for Intrinsic Phenotype Classification of Metastatic Castration‐Resistant Prostate Cancer,” Clinical Cancer Research 28 (2022): 3127–3140. 35552660 10.1158/1078-0432.CCR-21-4289 PMC9365375

[40] R. Aggarwal , J. Huang , J. J. Alumkal , et al., “Clinical and Genomic Characterization of Treatment‐Emergent Small‐Cell Neuroendocrine Prostate Cancer: A Multi‐Institutional Prospective Study,” Journal of Clinical Oncology: Official Journal of the American Society of Clinical Oncology 36 (2018): 2492–2503. 29985747 10.1200/JCO.2017.77.6880 PMC6366813

[41] H. I. Scher , R. P. Graf , N. A. Schreiber , et al., “Nuclear‐Specific AR‐V7 Protein Localization Is Necessary to Guide Treatment Selection in Metastatic Castration‐Resistant Prostate Cancer,” European Urology 71, no. 6 (2017): 874–882, 10.1016/j.eururo.2016.11.024. 27979426 PMC5401782

[42] M. Annala , G. Vandekerkhove , D. Khalaf , et al., “Circulating Tumor DNA Genomics Correlate With Resistance to Abiraterone and Enzalutamide in Prostate Cancer,” Cancer Discovery 8, no. 4 (2018): 444–457, 10.1158/2159-8290.CD-17-0937. 29367197

[43] Y. Yang , S. Wang , P. Ma , et al., “Drug Conjugate‐Based Anticancer Therapy—Current Status and Perspectives,” Cancer Letters 552 (2023): 215969. 36279982 10.1016/j.canlet.2022.215969

[44] W. Abida , J. Cyrta , G. Heller , et al., “Genomic Correlates of Clinical Outcome in Advanced Prostate Cancer,” Proceedings of the National Academy of Sciences of the United States of America 116 (2019): 11428–11436. 31061129 10.1073/pnas.1902651116 PMC6561293

[45] M. K. Buyyounouski , P. L. Choyke , J. McKenney , et al., “Prostate Cancer ‐ Major Changes in the American Joint Committee on Cancer Eighth Edition Cancer Staging Manual,” CA: A Cancer Journal for Clinicians 67 (2017): 245–253. 28222223 10.3322/caac.21391 PMC6375094

[46] E. Johansson , G. Steineck , L. Holmberg , et al., “Long‐Term Quality‐Of‐Life Outcomes After Radical Prostatectomy or Watchful Waiting: The Scandinavian Prostate Cancer Group‐4 Randomised Trial,” Lancet Oncology 12 (2011): 891–899. 21821474 10.1016/S1470-2045(11)70162-0

[47] C. W. Chua and M. Kruithof‐de Julio , “Exploring Prostate Cancer in the Post‐Genomic Era,” Cancer Letters 553 (2023): 215992. 36397638 10.1016/j.canlet.2022.215992

[48] H. Hieronymus , N. Schultz , A. Gopalan , et al., “Copy Number Alteration Burden Predicts Prostate Cancer Relapse,” Proceedings of the National Academy of Sciences of the United States of America 111 (2014): 11139–11144. 25024180 10.1073/pnas.1411446111 PMC4121784

[49] J.‐H. Kim , P. N. Bogner , S.‐H. Baek , et al., “Up‐Regulation of Peroxiredoxin 1 in Lung Cancer and Its Implication as a Prognostic and Therapeutic Target,” Clinical Cancer Research 14, no. 8 (2008): 2326–2333, 10.1158/1078-0432.CCR-07-4457. 18413821

[50] K.‐H. Chung , D. H. Lee , Y. Kim , et al., “Proteomic Identification of Overexpressed PRDX 1 and Its Clinical Implications in Ovarian Carcinoma,” Journal of Proteome Research 9 (2010): 451–457. 19902980 10.1021/pr900811x

[51] C. Dasari , K. R. K. Reddy , S. Natani , T. R. L. Murthy , S. Bhukya , and R. Ummanni , “Tumor Protein D52 (Isoform 3) Interacts With and Promotes Peroxidase Activity of Peroxiredoxin 1 in Prostate Cancer Cells Implicated in Cell Growth and Migration,” Biochimica et Biophysica Acta (BBA)—Molecular Cell Research 1866, no. 8 (2019): 1298–1309, 10.1016/j.bbamcr.2019.04.007. 30981892

[52] J. Rho , S. Qin , J. Y. Wang , and M. H. A. Roehrl , “Proteomic Expression Analysis of Surgical Human Colorectal Cancer Tissues: Up‐Regulation of PSB7, PRDX1, and SRP9 and Hypoxic Adaptation in Cancer,” Journal of Proteome Research 7 (2008): 2959–2972. 18549262 10.1021/pr8000892 PMC2693877

[53] E.‐Y. Lee , J.‐Y. Kang , and K.‐W. Kim , “Expression of Cyclooxygenase‐2, Peroxiredoxin I, Peroxiredoxin 6 and Nuclear Factor‐κB in Oral Squamous Cell Carcinoma,” Oncology Letters 10 (2015): 3129–3136. 26722300 10.3892/ol.2015.3705 PMC4665682

[54] J. Chuerduangphui , T. Ekalaksananan , C. Heawchaiyaphum , P. Vatanasapt , and C. Pientong , “Peroxiredoxin 2 Is Highly Expressed in Human Oral Squamous Cell Carcinoma Cells and Is Upregulated by Human Papillomavirus Oncoproteins and Arecoline, Promoting Proliferation,” PLoS One 15 (2020): e0242465. 33332365 10.1371/journal.pone.0242465 PMC7746188

[55] C.‐F. Huang , Z. J. Sun , Y. F. Zhao , X. M. Chen , J. Jia , and W. F. Zhang , “Increased Expression of Peroxiredoxin 6 and Cyclophilin A in Squamous Cell Carcinoma of the Tongue,” Oral Diseases 17 (2011): 328–334. 20796224 10.1111/j.1601-0825.2010.01730.x

[56] Z. Najafi , A. Mohamadnia , R. Ahmadi , et al., “Proteomic and Genomic Biomarkers for Non‐Small Cell Lung Cancer: Peroxiredoxin, Haptoglobin, and Alpha‐1 Antitrypsin,” Cancer Medicine 9 (2020): 3974–3982. 32232956 10.1002/cam4.3019 PMC7286458

[57] Y. Zhang , J. Jiang , J. Zhang , et al., “CircDIDO1 Inhibits Gastric Cancer Progression by Encoding a Novel DIDO1‐529aa Protein and Regulating PRDX2 Protein Stability,” Molecular Cancer 20, no. 1 (2021): 101, 10.1186/s12943-021-01390-y. 34384442 PMC8359101

[58] W. Wang , J. Wei , H. Zhang , et al., “PRDX2 Promotes the Proliferation of Colorectal Cancer Cells by Increasing the Ubiquitinated Degradation of p53,” Cell Death & Disease 12 (2021): 605. 34117220 10.1038/s41419-021-03888-1 PMC8196203

[59] M. Shiota , A. Yokomizo , E. Kashiwagi , et al., “Peroxiredoxin 2 in the Nucleus and Cytoplasm Distinctly Regulates Androgen Receptor Activity in Prostate Cancer Cells,” Free Radical Biology & Medicine 51 (2011): 78–87. 21539911 10.1016/j.freeradbiomed.2011.04.001

[60] P. Ramasamy , A.‐M. Larkin , A. Linge , et al., “PRDX3 Is Associated With Metastasis and Poor Survival in Uveal Melanoma,” Journal of Clinical Pathology 73 (2020): 408–412. 31771972 10.1136/jclinpath-2019-206173

[61] Z. Liu , Y. Hu , H. Liang , Z. Sun , S. Feng , and H. Deng , “Silencing PRDX3 Inhibits Growth and Promotes Invasion and Extracellular Matrix Degradation in Hepatocellular Carcinoma Cells,” Journal of Proteome Research 15, no. 5 (2016): 1506–1514, 10.1021/acs.jproteome.5b01125. 26983019

[62] Q. Wei , H. Jiang , Z. Xiao , et al., “Sulfiredoxin‐Peroxiredoxin IV Axis Promotes Human Lung Cancer Progression Through Modulation of Specific Phosphokinase Signaling,” Proceedings of the National Academy of Sciences of the United States of America 108 (2011): 7004–7009. 21487000 10.1073/pnas.1013012108 PMC3084097

[63] T. H. Kim , J. Song , S. R. Alcantara Llaguno , et al., “Suppression of Peroxiredoxin 4 in Glioblastoma Cells Increases Apoptosis and Reduces Tumor Growth,” PLoS One 7 (2012): e42818. 22916164 10.1371/journal.pone.0042818 PMC3419743

[64] M. J. López‐Grueso , D. J. Lagal , Á. F. García‐Jiménez , et al., “Knockout of PRDX6 Induces Mitochondrial Dysfunction and Cell Cycle Arrest at G2/M in HepG2 Hepatocarcinoma Cells,” Redox Biology 37 (2020): 101737. 33035814 10.1016/j.redox.2020.101737 PMC7554216

[65] H. Li , Z. Wu , R. Zhong , Q. Zhang , Q. Chen , and Y. Shen , “PRDX6 Knockout Restrains the Malignant Progression of Intrahepatic Cholangiocarcinoma,” Medical Oncology 39 (2022): 250. 36209344 10.1007/s12032-022-01822-9 PMC9547796

[66] A. Nicolussi , S. D'Inzeo , C. Capalbo , G. Giannini , and A. Coppa , “The Role of Peroxiredoxins in Cancer,” Molecular and Clinical Oncology 6 (2017): 139–153. 28357082 10.3892/mco.2017.1129 PMC5351761

[67] Y. Liu , P. Wang , W. Hu , and D. Chen , “New Insights Into the Roles of Peroxiredoxins in Cancer,” Biomedicine & Pharmacotherapy 164 (2023): 114896. 37210897 10.1016/j.biopha.2023.114896

[68] B. Knoops , J. Goemaere , V. Van der Eecken , and J.‐P. Declercq , “Peroxiredoxin 5: Structure, Mechanism, and Function of the Mammalian Atypical 2‐Cys Peroxiredoxin,” Antioxidants & Redox Signaling 15 (2011): 817–829. 20977338 10.1089/ars.2010.3584

[69] Y.‐Z. Jin , Y.‐X. Gong , Y. Liu , et al., “Peroxiredoxin V Silencing Elevates Susceptibility to Doxorubicin‐Induced Cell Apoptosis via ROS‐Dependent Mitochondrial Dysfunction in AGS Gastric Cancer Cells,” Anticancer Research 41 (2021): 1831–1840. 33813388 10.21873/anticanres.14949

[70] B. Kim , Y. S. Kim , H.‐M. Ahn , et al., “Peroxiredoxin 5 Overexpression Enhances Tumorigenicity and Correlates With Poor Prognosis in Gastric Cancer,” International Journal of Oncology 51 (2017): 298–306. 28535004 10.3892/ijo.2017.4013

[71] H.‐M. Ahn , J. W. Yoo , S. Lee , H. J. Lee , H. S. Lee , and D. S. Lee , “Peroxiredoxin 5 Promotes the Epithelial‐Mesenchymal Transition in Colon Cancer,” Biochemical and Biophysical Research Communications 487 (2017): 580–586. 28431931 10.1016/j.bbrc.2017.04.094

[72] A.‐C. Gérard , M.‐C. Many , C. Daumerie , B. Knoops , and I. M. Colin , “Peroxiredoxin 5 Expression in the Human Thyroid Gland,” Thyroid 15, no. 3 (2005): 205–209, 10.1089/thy.2005.15.205. 15785239

[73] M. Shiota , H. Izumi , N. Miyamoto , et al., “Ets Regulates peroxiredoxin1 and 5 Expressions Through Their Interaction With the High‐Mobility Group Protein B1,” Cancer Science 99, no. 10 (2008): 1950–1959, 10.1111/j.1349-7006.2008.00912.x. 19016754 PMC11159958

[74] R. Wang , Y. Mi , J. Ni , et al., “Identification of PRDX5 as A Target for the Treatment of Castration‐Resistant Prostate Cancer,” Advanced Science 11 (2024): e2304939. 38115765 10.1002/advs.202304939 PMC10916659

[75] S. S. Sabharwal and P. T. Schumacker , “Mitochondrial ROS in Cancer: Initiators, Amplifiers or an Achilles' Heel?,” Nature Reviews. Cancer 14 (2014): 709–721. 25342630 10.1038/nrc3803 PMC4657553

[76] L. Sainero‐Alcolado , J. Liaño‐Pons , M. V. Ruiz‐Pérez , and M. Arsenian‐Henriksson , “Targeting Mitochondrial Metabolism for Precision Medicine in Cancer,” Cell Death and Differentiation 29 (2022): 1304–1317. 35831624 10.1038/s41418-022-01022-y PMC9287557

[77] C. Glorieux , S. Liu , D. Trachootham , and P. Huang , “Targeting ROS in Cancer: Rationale and Strategies,” Nature Reviews. Drug Discovery 23 (2024): 583–606. 38982305 10.1038/s41573-024-00979-4

[78] B. Wallden , J. Storhoff , T. Nielsen , et al., “Development and Verification of the PAM50‐Based Prosigna Breast Cancer Gene Signature Assay,” BMC Medical Genomics 8 (2015): 54. 26297356 10.1186/s12920-015-0129-6 PMC4546262

