# Supplementary figures and images for: Characterization of the Biochemical Recurrence Prediction Ability and Progression Correlation of Peroxiredoxins Family in Prostate Cancer Based on Integrating Single‐Cell RNA‐Seq and Bulk RNA‐Seq Cohorts
Source: Cancer Med. 2025 Apr 25;14(9):e70855. doi: 10.1002/cam4.70855 (PMC12031674; doi:10.1002/cam4.70855)

**GSE141445**

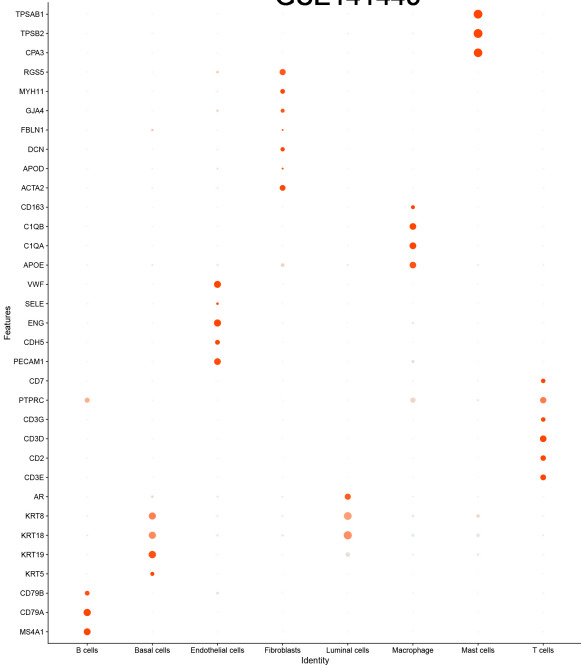

**GSE157703**

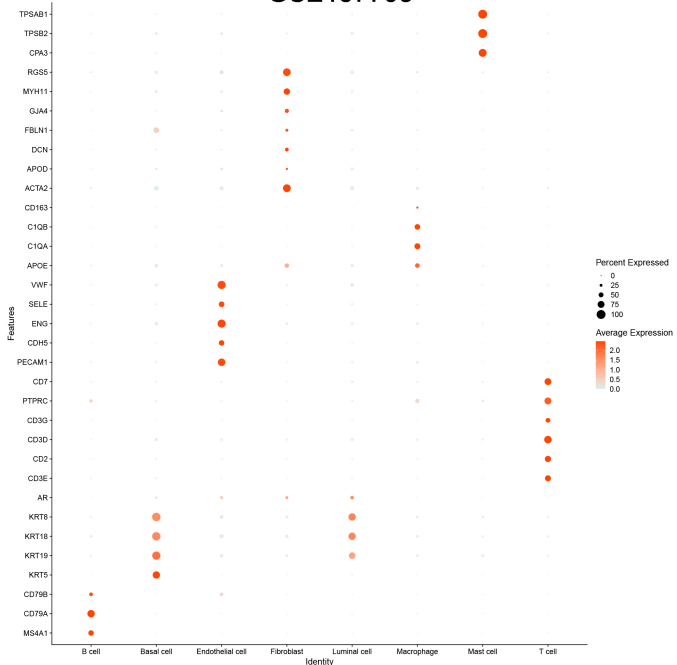

Supplement: Supplementary file 1 — Figure S1. Bubble heatmap showing marker genes of eight major cell types in GSE141445 and GSE157703 dataset. Dot size indicates fraction of expressing cells, and represents to expression levels. [file CAM4-14-e70855-s003.pdf]

A

GSE141445

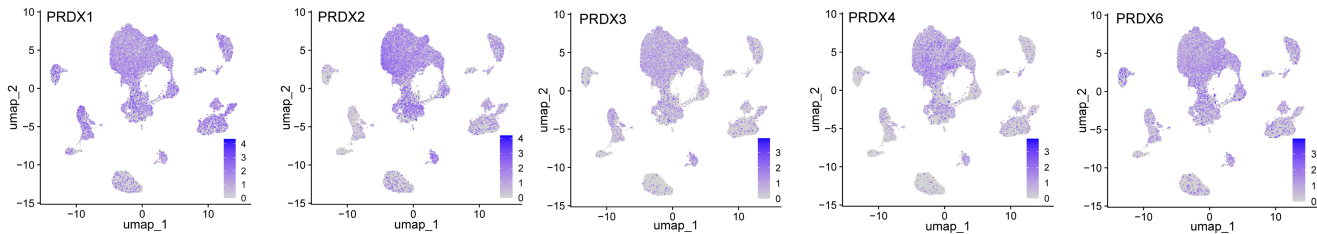

B

GSE157703

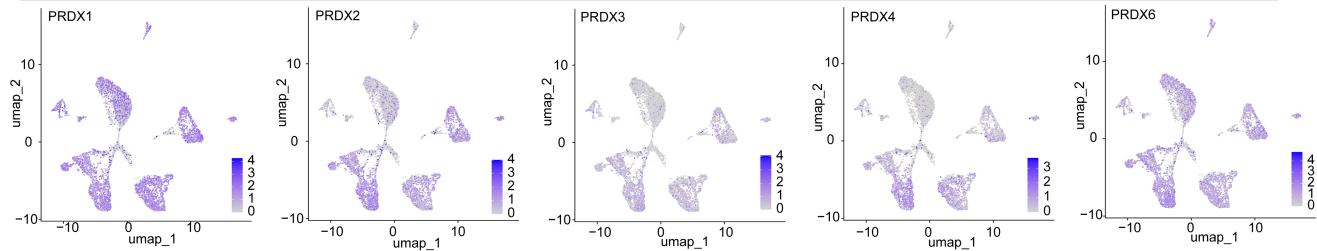

Supplement: Supplementary file 2 — Figure S2. Depicting the expression of rest 5 genes in single‐cell sequencing with uniform manifold approximation and projection (UMAP) using GSE141445 and GSE157703 database. [file CAM4-14-e70855-s002.pdf]

A

## Cambridge

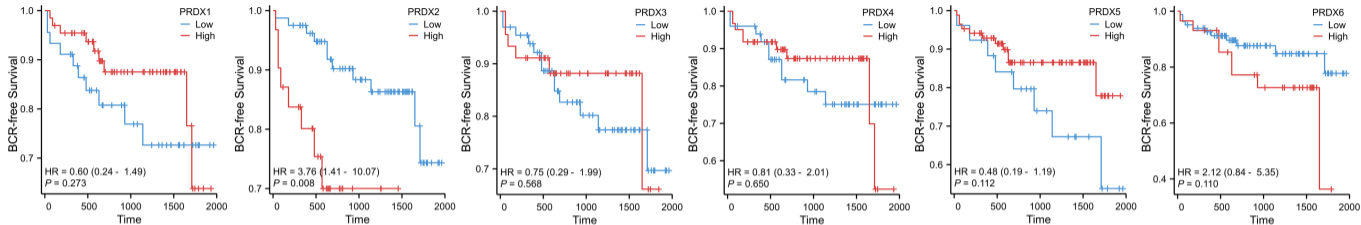

B

## GSE116918

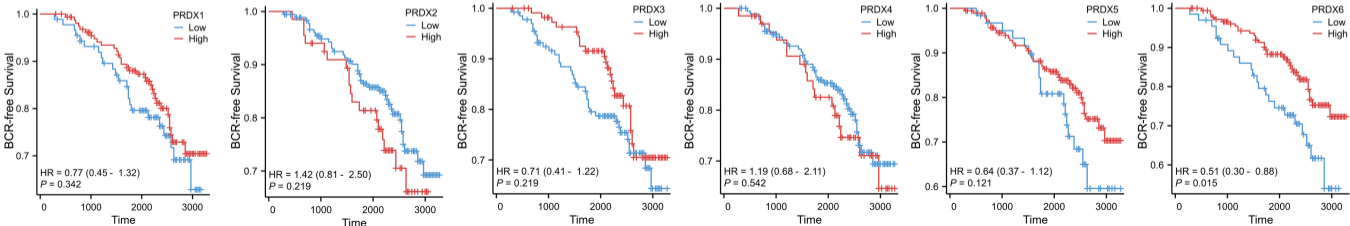

Supplement: Supplementary file 4 — Figure S4. Kaplan–Meier curves illustrate the BCR status of prostate cancer patients across Cambridge (A) and GSE116918 (B) database. [file CAM4-14-e70855-s004.pdf]

# A

## GS≥7

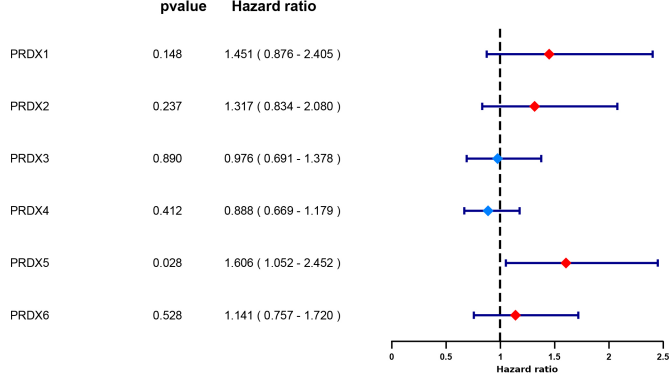

# B

## N1

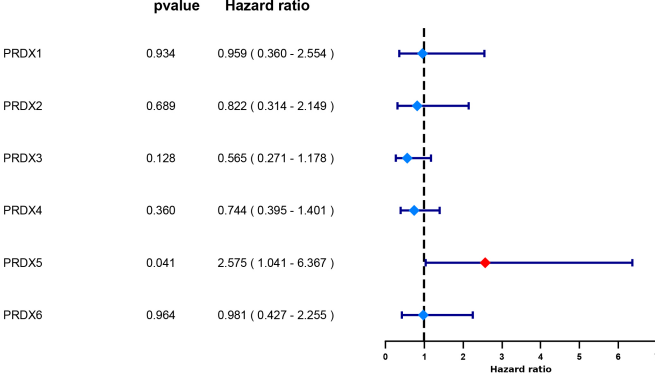

# C

## N0

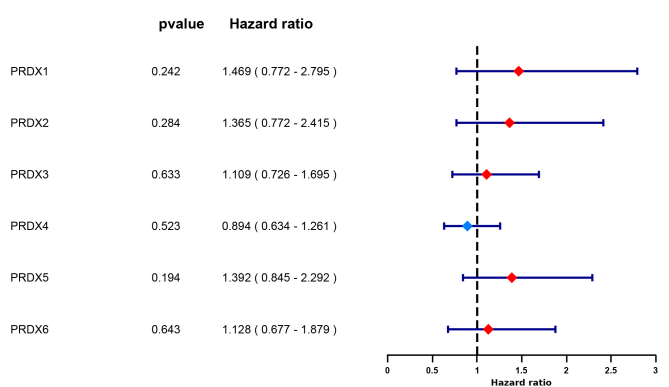

# D

## Pathological stage ≥T3

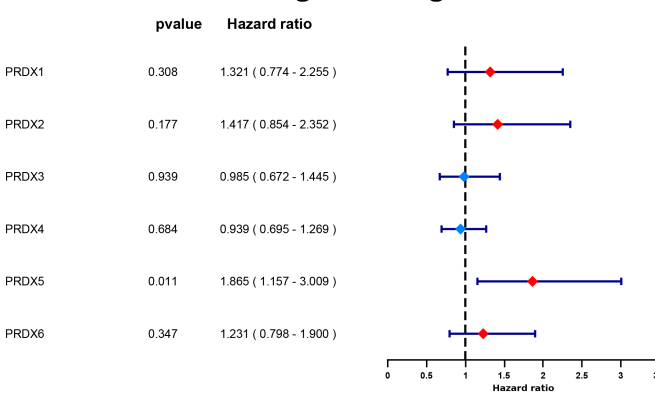

# E

## Pathological stage <T3

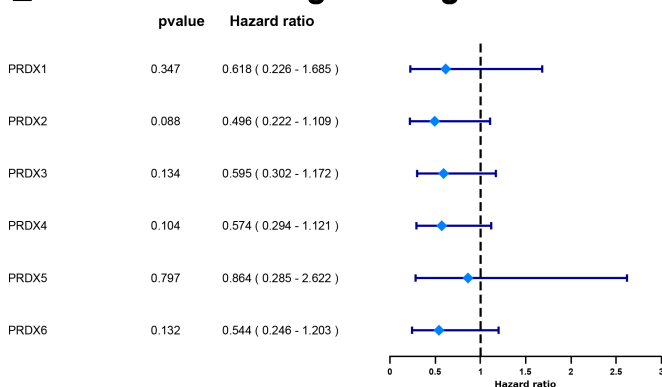

Supplement: Supplementary file 5 — Figure S5. (A) Univariate analyses of PRDXs in prostate cancer patients with Gleason score ≥ 7 from TCGA. (B) Univariate analyses of PRDXs in prostate cancer patients with clinical N1 stage from TCGA. (C) Univariate analyses of PRDXs in prostate cancer patients with clinical N0 stage from TCGA. (D) Univariate analyses of PRDXs in prostate cancer patients with pathological T stage ≥ 3 from TCGA. (E) Univariate analyses of PRDXs in prostate cancer patients with pathological T stage < 3 from TCGA. [file CAM4-14-e70855-s006.pdf]

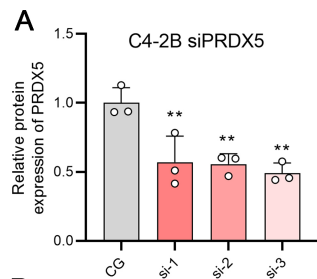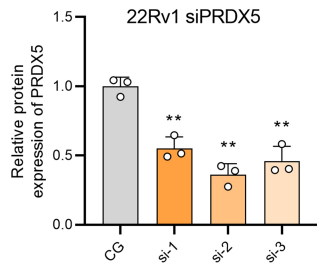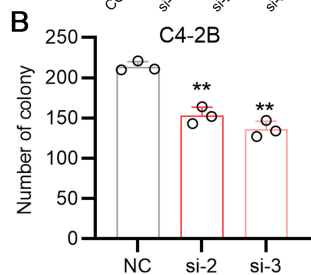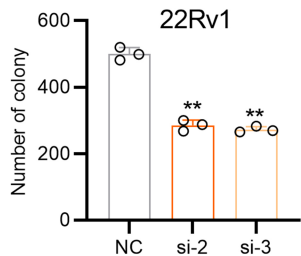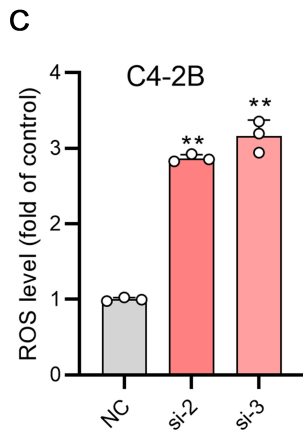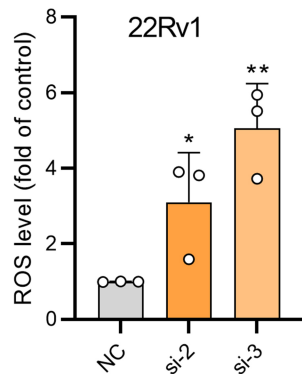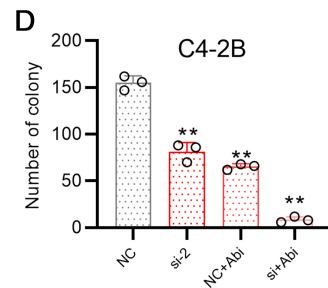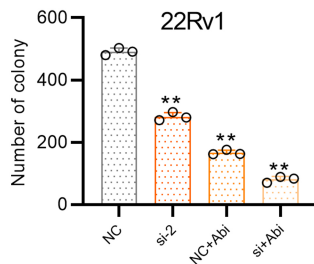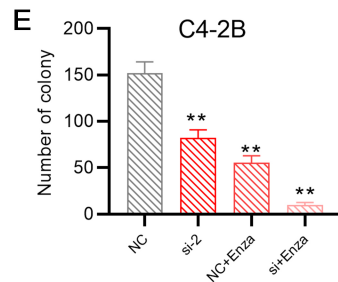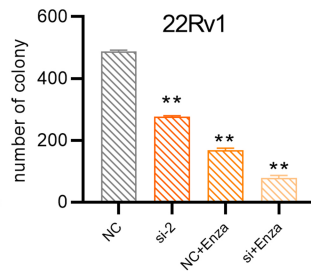

Supplement: Supplementary file 6 — Figure S6. (A) Protein quantification assay for western blot experiment in Figure 10A. (B) Statistics analysis of colony formation assay in Figure 10B. (C) Statistics analysis of ROS flow cytometry assay in Figure 10F. (D) Statistics analysis of colony formation assay in Figure 10I. (E) Statistics analysis of colony formation assay in Figure 10K (*p < 0.05, **p < 0.01). [file CAM4-14-e70855-s001.pdf]
