# Supplementary material for: Characterization of the Biochemical Recurrence Prediction Ability and Progression Correlation of Peroxiredoxins Family in Prostate Cancer Based on Integrating Single‐Cell RNA‐Seq and Bulk RNA‐Seq Cohorts
Source: Cancer Med. 2025 Apr 25;14(9):e70855. doi: 10.1002/cam4.70855 (PMC12031674; doi:10.1002/cam4.70855)
Supplement: Supplementary file 3 — Figure S3. Immunohistochemistry staining of the rest 5 genes in Human protein altas database. [file CAM4-14-e70855-s005.pdf]

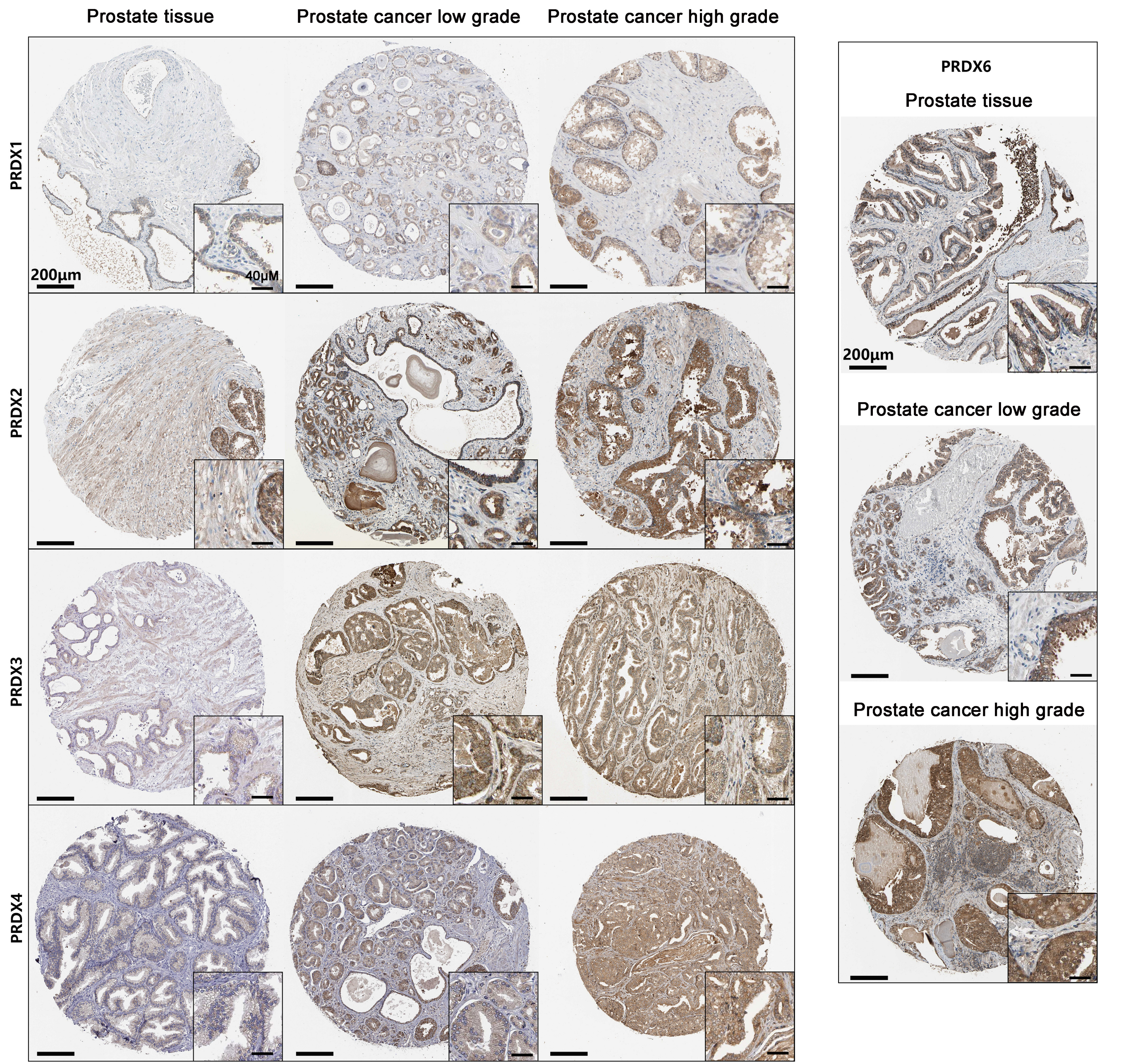
Prostate tissue
Prostate cancer low grade
Prostate cancer high grade
PRDX1
PRDX2
PRDX3
PRDX4
200µm
40µM
PRDX6
Prostate tissue
Prostate cancer low grade
Prostate cancer high grade
200µm
